# A tumor microenvironment-based prognostic index for osteosarcoma

**DOI:** 10.1186/s12929-023-00917-3

**Published:** 2023-04-13

**Authors:** Changwu Wu, Siming Gong, Yingjuan Duan, Chao Deng, Sonja Kallendrusch, Laura Berninghausen, Georg Osterhoff, Nikolas Schopow

**Affiliations:** 1 Institute of Anatomy, University of Leipzig, Liebigstraße 13, 04103 Leipzig, Germany; 2 Faculty of Chemistry and Mineralogy, University of Leipzig, 04103 Leipzig, Germany; 3 Department of Orthopedics, Xiangya Hospital, Central South University, Changsha, 410008 China; 4 Faculty of Medicine, Health and Medical University Potsdam, 14471 Potsdam, Germany; 5 Department of Orthopedics, Trauma and Plastic Surgery, Sarcoma Center, University Hospital Leipzig, 04103 Leipzig, Germany

**Keywords:** Osteosarcoma, Tumor microenvironment, Prognostic index, Checkpoint inhibitor therapy, Immune cell infiltration, Big data

## Abstract

**Background:**

The tumor microenvironment (TME) has a central role in the oncogenesis of osteosarcomas. The composition of the TME is essential for the interaction between tumor and immune cells. The aim of this study was to establish a prognostic index (TMEindex) for osteosarcoma based on the TME, from which estimates about patient survival and individual response to immune checkpoint inhibitor (ICI) therapy can be deduced.

**Methods:**

Based on osteosarcoma samples from the Therapeutically Applicable Research to Generate Effective Treatments (TARGET) database, the ESTIMATE algorithm was used to estimate ImmuneScore and StromalScore. Combined differentially expressed gene analysis, weighted gene co-expression network analyses, the Least Absolute Shrinkage and Selection Operator regression and stepwise regression to construct the TMEindex. The prognostic role of TMEindex was validated in three independent datasets. The molecular and immune characteristics of TMEindex and the impact on immunotherapy were then comprehensively investigated. The expression of TMEindex genes in different cell types and its effects on osteosarcoma cells were explored by scRNA-Seq analysis and molecular biology experiments.

**Results:**

Fundamental is the expression of MYC, P4HA1, RAMP1 and TAC4. Patients with high TMEindex had worse overall survival, recurrence-free survival, and metastasis-free survival. TMEindex is an independent prognostic factor in osteosarcoma. TMEindex genes were mainly expressed in malignant cells. The knockdown of MYC and P4HA1 significantly inhibited the proliferation, invasion and migration of osteosarcoma cells. A high TME index is related to the MYC, mTOR, and DNA replication-related pathways. In contrast, a low TME index is related to immune-related signaling pathways such as the inflammatory response. The TMEindex was negatively correlated with ImmuneScore, StromalScore, immune cell infiltration, and various immune-related signature scores. Patients with a higher TMEindex had an immune-cold TME and higher invasiveness. Patients with a low TME index were more likely to respond to ICI therapy and achieve clinical benefit. In addition, the TME index correlated with response to 29 oncologic drugs.

**Conclusions:**

The TMEindex is a promising biomarker to predict the prognosis of patients with osteosarcoma and their response to ICI therapy, and to distinguish the molecular and immune characteristics.

**Supplementary Information:**

The online version contains supplementary material available at 10.1186/s12929-023-00917-3.

## Introduction

Osteosarcoma, the earliest known hominin cancer [1], is commonly found in children and young adults [2, 3]. Although osteosarcoma is the most common primary malignant bone tumor, it remains rare with only 800 to 900 cases diagnosed annually in the United States [4]. The current 5-year survival rate for patients with localized osteosarcoma is about 60%, and when recurrences or metastases occur, it is only 20% [5]. Unlike the rapid advances achieved in the treatment of other solid tumors, outcomes for patients with osteosarcoma have not significant improved over the past three decades [4, 6]. The reasons for this discrepancy are multifaceted and include a missing understanding of the genetic complexity of osteosarcoma [4]. There is a lack of clarity regarding the prognosis-related mechanisms of osteosarcoma [7], and accurate assessment of the prognosis of individual patients becomes extremely difficult due to its tumor heterogeneity [8]. However, because patients with osteosarcoma are often extremely young, there is a great need to further improve the prognosis of patients with osteosarcoma to achieve long-term survival in young patients. Continued research into new biomarkers and therapeutic strategies is essential to predict prognosis and to improve individualized treatment. In addition, current directions to further improve the prognosis of patients with osteosarcoma lie mainly in identifying effective risk stratification algorithms to minimize treatment toxicities and iatrogenic secondary tumors while improving therapy benefits [9].

As the understanding of osteosarcoma deepens, oncologists are increasingly recognizing the difficulty of targeting osteosarcoma cells alone to further improve patient outcomes, and thus the focus is gradually being shifted to the tumor microenvironment (TME) of osteosarcoma [10]. TME, the environment in which the tumor resides, consists of malignant cells, immune cells, stromal cells, extracellular matrix, and a variety of cytokines [11, 12]. A large body of evidence suggests that TME has a non-negligible role in tumorigenesis, proliferation, metastasis, and drug resistance acquisition in osteosarcoma [13–15], thereby affecting the prognosis of patients with osteosarcoma. In addition, low immune cell infiltration levels in TME not only represent lower anti-tumor immunity, but also help tumor cells to evade immune attacks [16, 17]. Stromal cells, including cancer-associated fibroblast, then exert a direct immunosuppressive effect [17, 18]. This suggests that the TME status may be a potential prognostic biomarker as well as a predictive marker for the response to treatments such as immunotherapy in osteosarcoma.

Although the TME status may be a promising biomarker for patients with osteosarcoma, there is a lack of effective methods to clarify TME component in osteosarcoma. In this study, by integrating extensive transcriptome sequencing data from the Therapeutically Applicable Research to Generate Effective Treatments (TARGET) and the Gene Expression Omnibus (GEO) databases for comprehensive analysis to identify key TME-related genes, an osteosarcoma TME-based prognostic index (TMEindex) was developed to quantify the status of TME. This quantitative molecular signature of TME is not only an intuitive and effective prognostic prediction tool, but also exhibits effective predictive performance for immunotherapeutic response.

## Methods

### Datasets, tissue samples, and cell lines

Standardized RNA-seq data and clinical information for 84 osteosarcoma samples from the TARGET database were obtained from the UCSC Xena browser (http://xena.ucsc.edu/). Together 140 samples with normalized RNA-seq or microarray data were obtained from the GEO database (GSE21257 [19], GSE16091 [20], and GSE33382 [21]) (https://www.ncbi.nlm.nih.gov/geo/). Gene expression data for the TARGET and three GEO cohorts were transformed by log2(x + 1), and detailed clinical information for the four cohorts is summarized in Additional file 1: Table S1. In addition, a standardized pan-cancer dataset was obtained from the UCSC Xena browser, which included 9798 samples for 37 cancers from The Cancer Genome Atlas (TCGA) and TARGET databases. Updated prognostic information for TCGA cohort was also obtained from a previous study [22]. To explore the prognostic value of the TMEindex in patients receiving immunotherapy, two anti-PD-L1 cohorts, including the IMvigor210-Bladder urothelial carcinoma (BLCA) and IMvigor210-Kidney cancer cohorts, were extracted from the study by Mariathasan et al. [23]. For the immunotherapy cohorts, detailed gene expression data and clinical annotations are available from IMvigor210 Core Biologies (http://research-pub.gene.com/IMvigor210CoreBiologies) based on the Creative Commons 3.0 license. For the raw count data, it was normalized and converted to transcripts per kilobase million (TPM) values by the R package “preprocessCore”, and then a log2(x + 1) transformation was performed.

A single-cell RNA-sequencing (scRNA-seq) cohort containing 11 osteosarcoma samples was obtained from the GEO database (GSE152048) [24]. Quality control and downstream analysis are performed according to the standard process of the R package “Seurat” (v.4.3.0). Low quality cells (< 3 genes/cell and > 20% mitochondrial genes) and genes expressed in less than three cells were filtered out. The housekeeping and mitochondrial genes were then filtered out, and finally, 26,175 genes and 123,322 cells were included in the study. After performing standard data downscaling and clustering, clusters were annotated using previously reported cellular markers [24].

Fourteen samples of osteosarcoma tissues and five samples of normal tissues were collected from patients who underwent surgical resection at the Department of Orthopedics, Xiangya Hospital, Central South University, Hunan, China in 2018–2019. All samples were sent to the Department of Pathology for pathological evaluation after surgical resection, and the remaining samples were preserved in paraffin wax. All patients have completed follow-up information, and since most patients are still alive, only relapse information is currently available. This study was approved by the Medical Ethics Committee of Xiangya Hospital of Central South University (Approval number: 202303046).

The osteosarcoma cell lines MG-63 and U2OS were obtained from the Xiangya cell repository. The cells were cultured in Dulbecco’s modified Eagle’s medium (DMEM, Biological Industries, Israel) containing antibiotics and 10% fetal bovine serum (Gibco, USA) at 5% CO_2_ and 37 °C. The small interfering RNAs (siRNAs) of MYC, P4HA1, RAMP1 and TAC4 and the empty vector (si-NC) were synthesized by GenePharma (Shanghai, China). Cell transfection was performed with Lipofectamine^®^ 3000 (Invitrogen/Thermo Fisher Scientific, Carlsbad, CA, USA) per the manufacturers instructions.

### Construction of the TME-based prognostic index

The TARGET cohort was used for the development of the TMEindex. First, ImmuneScore and StromalScore were calculated for each sample using the ESTIMATE algorithm [17], which quantifies immune and stromal cells based on molecular markers. All samples were divided into two groups using the median ImmuneScore or StromalScore, and differentially expressed genes (DEGs) were calculated between the high and low ImmuneScore/StromalScore groups based on the R package "limma" with false discovery rate (FDR) < 0.05 and |Fold Change (FC)|> 1.5 as thresholds. Subsequently, gene modules closely associated with ImmuneScore and StromalScore were further identified using the unsigned weighted gene co-expression network analysis (WGCNA) based on the R package “WGCNA” [25]. The optimal soft threshold in WGCNA was determined based on the scale-free network and the mean connectivity. Gene modules with |correlation coefficients|> 0.5 were identified as TME-related gene modules. All module genes associated with ImmuneScore and StromalScore were extracted, respectively and intersected with the corresponding DEGs, and then Venn diagrams were plotted. The intersection of DEGs with TME-related module genes were considered as reliable immune-related or stromal-related genes. The Least Absolute Shrinkage and Selection Operator (LASSO) regression can be combined with the Cox model for accurate screening of survival-related biomarkers [26]. Identified immune and stromal-related genes were, respectively, input into the LASSO regression for further downscaling and identification of hub genes. Ultimately, all immune-related and stromal-related hub genes were input into the stepwise Cox regression to construct the TMEindex, and the model with the largest C-index was determined to be the best model. The risk model was calculated using the following formula:

$$TMEindex=\sum {\beta }_{j}\times {Exp}_{i},$$

where β_j_ is the coefficient of each gene in the final risk model, and Exp_i_ is the gene expression value.

### Functional and pathway enrichment analysis

To explore TMEindex-related functions and pathway enrichment, the gene set enrichment analysis (GSEA) was performed using the GSEA software (Version 4.1.0) [27]. Base on the "c2.cp.kegg.v7.4.symbols" gene set and the HALLMARK gene set from the MSigDB database, different pathways and molecular mechanisms in the high and low TMEindex groups were evaluated. The minimum gene set was set to 5, the maximum gene set was set to 5000, and resamples were 1000. In addition, DEGs between high and low TMEindex groups were identified based on the R package "limma", and the threshold was set as *P* < 0.05 and |FC|> 1.5. Gene Ontology (GO) and Kyoto Encyclopedia of Genes and Genomes (KEGG) analysis of DEGs was then performed using the R package “clusterProfiler” [28]. To explore the correlation between TMEindex and other known core biological processes, a series of gene sets curated by Mariathasan et al. were used [23], which included a total of 18 important gene signatures including CD8 T-effector signature, antigen processing machinery, DNA replication, pan-fibroblast TGFβ response signature (Pan-F-TBRS) et al. In addition, the correlation between TMEindex and different immune-related biological processes were explored based on a study by Zeng et al. [29], including immune activation-relevant gene set, immune checkpoint-relevant gene set, and transforming growth factor (TGF)β/epithelial–mesenchymal transition (EMT) pathway-relevant gene set. *P* value of < 0.05 was considered statistically significant.

### Estimation of TME cell infiltration

The single-sample gene-set enrichment analysis (ssGSEA) method was used to quantify the relative abundance of different TME-infiltrating cell in each sample based on the R package “GSVA”. The gene sets used to mark different immune cells were derived from a study of Charoentong et al. [30], in which 28 human immune cell subtypes were stored.

### Association analysis of TMEindex and drug sensitivity

The standardized gene expression matrix of 809 tumor cell lines from the Genomics of Drug Sensitivity in Cancer (GDSC) database and the half-maximal inhibitory concentration (IC50) values of 189 drugs corresponding to the cell lines were used as a training set [31], then the oncoPredict algorithm was used to predict the IC50 value of every drug in individual osteosarcoma patients as we described previously [32, 33]. Subsequently, Spearman correlation analysis was used to calculate the correlation between TMEindex and drug sensitivity, the |correlation coefficient (R value)|> 0.3 and *P* < 0.01 was considered as a significant correlation.

### CCK-8 assay

Cell proliferation activity was measured using the CCK-8 kit (Dojindo Laboratories, Kumamoto, Japan). Transfected cells were cultivated for 24 h and then inoculated in 96-well plates with 2000 cells per well. After cell walling, 10 µl of CCK-8 reagent was added every 24 h to the wells for the desired assay. The absorbance was measured at 450 nm after 3 h of incubation at 37 °C.

### Transwell assay

The invasiveness of the cells was detected by Transwell assay. 2 × 10^4^ transfected U2OS and MG-63 cells were inoculated in the upper chamber and incubated in 5% serum medium, and the lower chamber was incubated in 15% serum medium. After 24 h of incubation, the stromal gel was wiped off the upper chamber with a cotton swab, and then the cells on the chamber membrane were fixed with 4% paraformaldehyde and stained with 10% crystal violet. Five random fields of view were counted for each chamber.

### Wound healing assay

Cell migration ability was detected using a wound healing assay (scratch assay). Transfected and untransfected cells were inoculated as a monolayer in a 6-well plate and incubated for 24 h. The monolayer of cells was scratched with a 100 μl pipette tip and the cells were washed with medium to remove cell debris. Scratch healing was observed using a microscope after 36 h of incubation.

### Immunohistochemistry (IHC)

IHC was carried out as described previously [34]. The following antibody was used: rabbit polyclonal antibody to MYC (Sangon Biotech, D155013, 1:200 dilution), rabbit polyclonal antibody to P4HA1 (Invitrogen, PA5-31246, 1:200 dilution), rabbit polyclonal antibody to RAMP1 (Invitrogen, PA5-50253, 1:50 dilution). Two pathologists, who did not know the identity of the samples, independently scored the staining results based on staining intensity and percentage of positive cells. Intensity was scored as follows: 0 (negative), 1 (weakly positive), 2 (moderately positive), and 3 (strongly positive). The percentage of positive cells was scored as follows: 0 (0%), 1 (1–25%), 2 (26–50%) and 3 (> 50%). The IHC score was the sum of the intensity score and the score of the percentage of positive cells.

### Statistical analysis

All statistical calculations were performed using R software (Version 4.1.2) and GraphPad Prism 9 (La Jolla, CA, USA). Differences between two groups were compared using unpaired Student's t-test or Wilcoxon rank sum test. Correlations between TMEindex and TME-infiltrating cells or biological pathways were calculated using Spearman’s correlation analysis. The R packages “survival” and “survminer” were used to calculate the correlation between the variables and survival time and to find the best cutoff value. Survival curves were generated using the Kaplan–Meier (KM) method, and significance was determined using the log-rank test. Univariate Cox regression analysis was used to calculate the prognostic significance and hazard ratios (HR) for TMEindex in pan-cancer. Multivariate Cox regression analysis was used to determine whether TMEindex was an independent prognostic factor using age and gender as covariates. The R package “pROC” was used to plot receiver operating characteristic (ROC) curves to verify the validity of the model and obtain the area under the curve (AUC). *p* < 0.05 was considered statistically significant, and unless otherwise stated, p values were two-sided.

## Results

### Relationship between ImmuneScore/StromalScore and clinical features and survival

The setup of this study is shown in Additional file 1: Fig. S1. ImmuneScore and StromalScore were calculated for each osteosarcoma in the TARGET cohort. The ImmuneScores in these patients were ranging from − 1558.1 to 2404.6 and StromalScores were ranging from − 797.2 to 1807.5. There was a significant positive correlation between ImmuneScore and StromalScore (Additional file 1: Fig. S2A). Age or gender had no significant effect on the ImmuneScore and StromalScore (Additional file 1: Fig. S2B, C). Survival analysis showed that patients with both a high ImmuneScore or a high StromalScore had a significant better overall survival (OS) and relapse-free survival (RFS) compared to patients with low scores (Additional file 1: Fig. S2D, E). The AUCs of the ImmuneScore were 0.66, 0.67 and 0.59 at 1, 3, and 5-year of OS, and 0.66, 0.62 and 0.54 at 1, 3, and 5-year of RFS (Additional file 1: Fig. S2D, E). The AUCs of the StromalScore were 0.69, 0.69 and 0.63 at 1, 3, and 5-year of OS, and 0.78, 0.60 and 0.59 at 1, 3, and 5-year of RFS (Additional file 1: Fig. S2F, G).

### Construction of the TME-based prognostic index

**Fig. 1:**
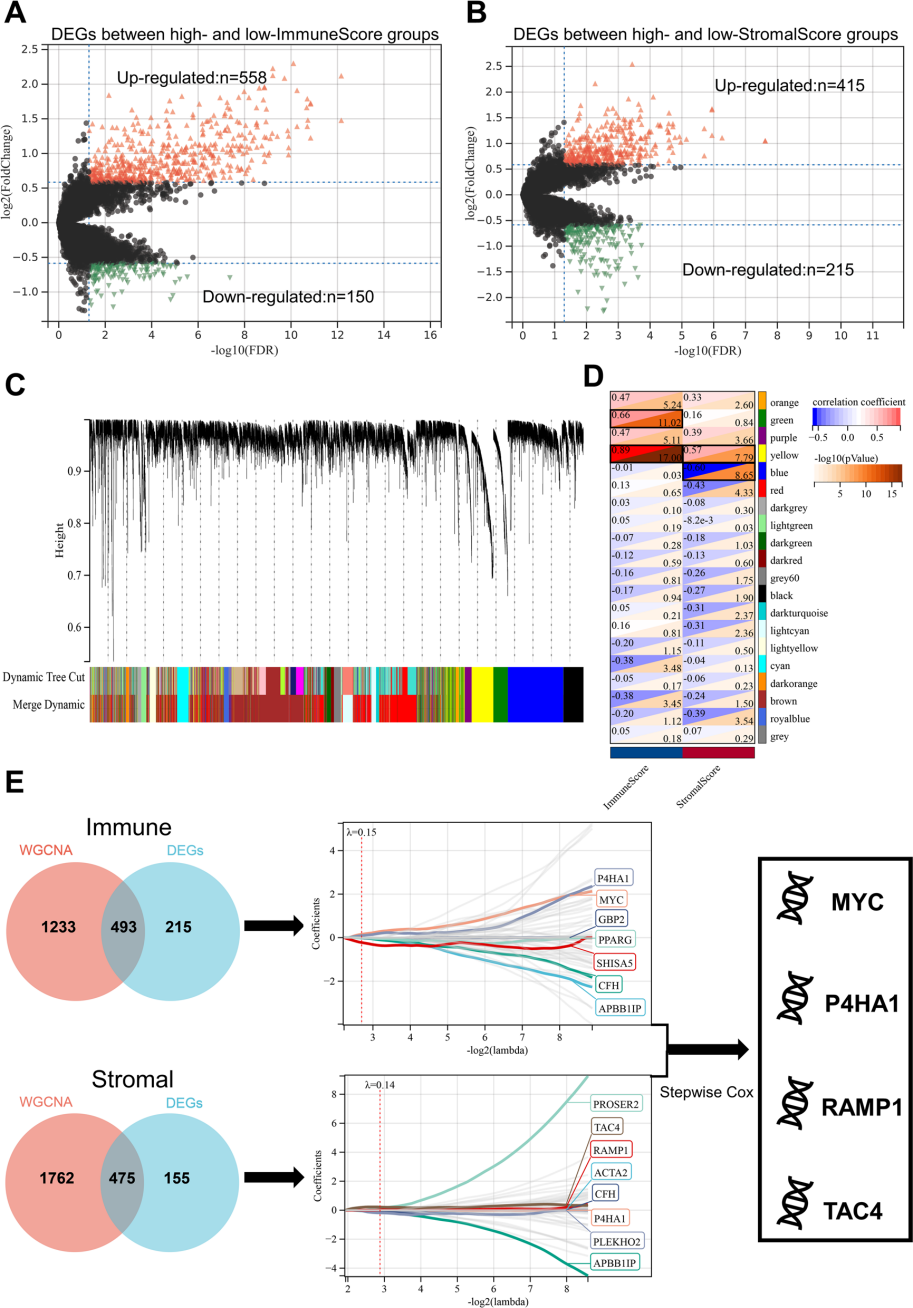
Construction of the TMEindex.** A** Volcano plot of differentially expressed genes between high-ImmuneScore group and low-ImmuneScore group. **B** Volcano plot of differentially expressed genes between high-StromalScore group and low-StromalScore group. **C** Gene modules identified by WGCNA. **D** Correlation analysis between gene modules and ImmuneScore/ StromalScore. Strongly correlated modules (|correlation coefficients|> 0.6, P < 0.05) are marked with black frames. **E** LASSO regression and stepwise regression for determining the final model. Venn plot shows the number of intersection genes between WGCNA and DEG analysis. These genes were further input into LASSO regression. The Y-axis shows LASSO coefficients and the X-axis is − log2(lambda). The genes obtained from LASSO regression downscaling were further input into stepwise regression to determine the TMEindex

Divided osteosarcoma patients in the TARGET cohort into high and low ImmuneScore/StromalScore groups using median as cut-off value, volcano plots indicated that 558 DEGs were upregulated and 150 DEGs were downregulated in the high ImmuneScore group (Fig. 1A; Additional file 2: Table S2), while 415 DEGs were upregulated and 215 DEGs were downregulated in the high StromalScore group (Fig. 1B; Additional file 2: Table S3). Subsequently, in WGCNA, the optimal soft threshold was determined as 5 based on scale-free and mean connectivity network maps (Additional file 1: Fig. S3), and 20 co-expressed gene modules were identified after merging modules with distances less than 0.25 (except for the grey module, which contains genes that cannot be classified) (Fig. 1C). Based on correlation analysis, the green and yellow modules were found to be strongly positively correlated with ImmuneScore, while the blue module was strongly negatively correlated with StromalScore and the yellow module was positively correlated with StromalScore (Fig. 1D; Additional file 2: Table S4). Venn plots showed the intersection of DEGs and TME-related gene modules in WGCNA, where 493 genes were identified as ImmuneScore-related genes and 475 genes were identified as StromalScore-related genes (Fig. 1E), These genes were further input into LASSO regression analysis for downscaling, and further screened for APBB1IP, CFH, GBP2, MYC, P4HA1, PPARG and SHISA5 in ImmuneScore-related, and screened for ACTA2, APBB1IP, CFH, P4HA1, PLEKHO2, PROSER2, RAMP1 and TAC4 in StromalScore-related genes (Fig. 1E). Finally, the APBB1IP, ACTA2, CFH, GBP2, MYC, P4HA1, PPARG, PLEKHO2, P4H, RAMP1, TAC4, and SHISA5 were input into stepwise regression to determine the optimal model, which we termed as TMEindex (Fig. 1E). The optimal model contains MYC, P4HA1, RAMP1, and TAC4, the C-index of the optimal model is 0.825. The model was: TMEindex = 0.8115 × MYC + 0.5565 × P4HA1 + 0.4634 × RAMP1 + 0.3422 × TAC4.

Further, the prognostic value of MYC, P4HA1, RAMP1 and TAC4 was also evaluated. Univariate Cox regression showed that these genes were strongly associated with OS in osteosarcoma (Additional file 1: Fig. S4A). KM curves indicated that patients with high expression of MYC, P4HA1, RAMP1 and TAC4 had significantly worse OS (Additional file 1: Fig. S4B–E) and RFS (Additional file 1: Fig. S5A–D).

### Prognostic predictive value of the TMEindex in the TARGET cohort

**Fig. 2:**
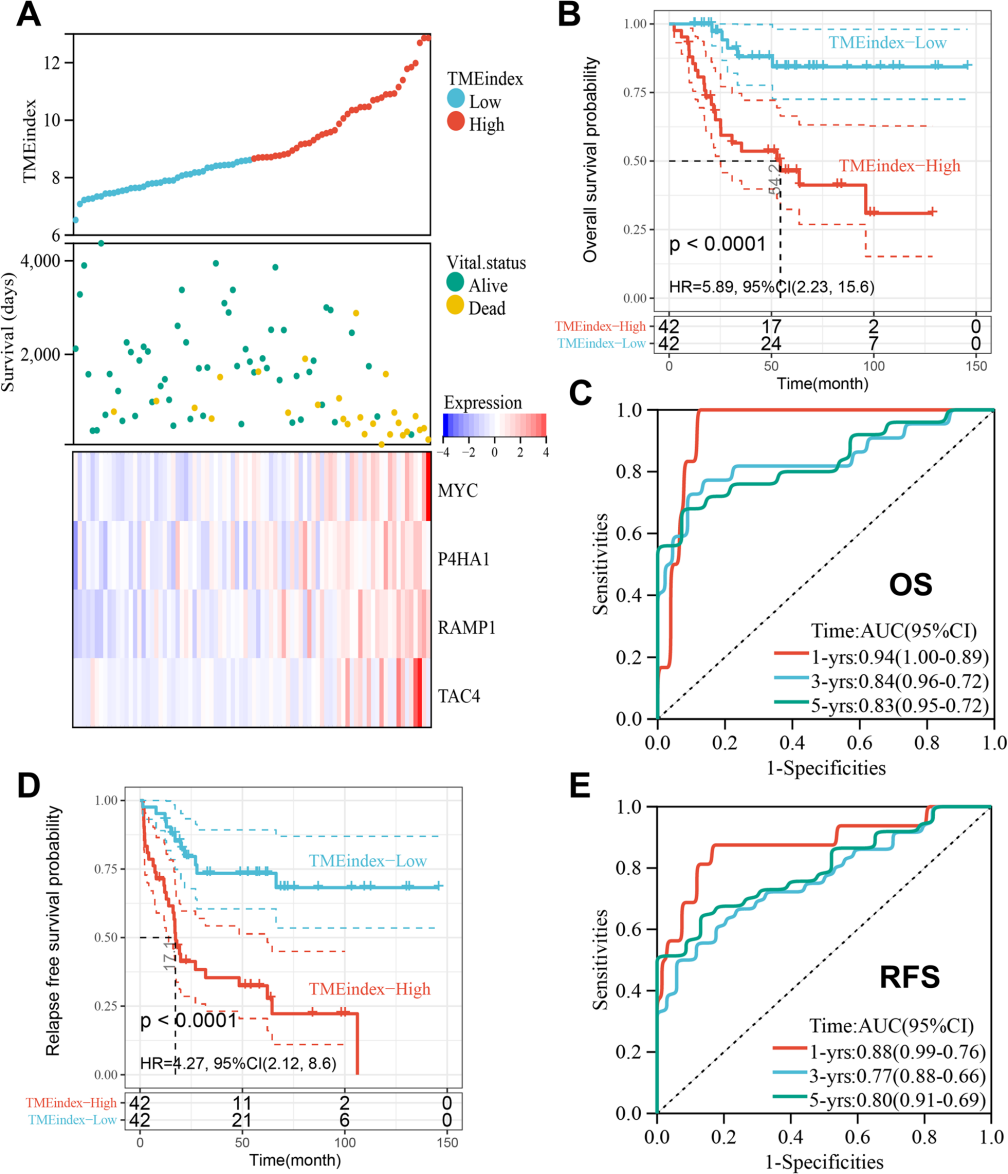
Prognostic predictive role of TMEindex.** A** The distribution of TMEindex and survival status and the heatmap of 4 genes of the TMEindex in the TARGET cohort. **B** Kaplan–Meier curve depicts the OS difference between TMEindex-high and TMEindex-low groups (log-rank P < 0.0001) in the TARGET cohort. Red representing the TMEindex-high group and blue representing the TMEindex-low group. **C** ROC curve showing the OS prediction efficiency of the TMEindex in the TARGET cohort. **D** Kaplan–Meier curve depicts the RFS difference between TMEindex-high and TMEindex-low groups (log-rank P < 0.0001) in the TARGET cohort. Red representing the TMEindex-high group and blue representing the TMEindex-low group. **E** ROC curve showing the RFS prediction efficiency of the TMEindex in the TARGET cohort

In the TARGET cohort, all patients were divided into high and low TMEindex groups using the median TMEindex (TMEindex = 8.615) as the risk threshold. Figure 2A showed the distribution of TMEindex and patients, the high TMEindex group had significantly shorter survival time and higher expression of MYC, P4HA1, RAMP1 and TAC4 than the low TMEindex group (Additional file 1: Fig. S6A, B). The KM curve shows that patients in the high TMEindex group had a significantly shorter OS (*P* < 0.0001; Fig. 2B). The AUCs of the TMEindex were 0.94, 0.84 and 0.83 at 1, 3, and 5-year of OS, respectively (Fig. 2C). As shown in Fig. 2D, patients with high TMEindex also had a shorter RFS than patients with low TMEindex (*P* < 0.0001). The AUCs of 1, 3, and 5-year RFS were 0.88, 0.77, 0.80 (Fig. 2E).

A stratified analysis was conducted to further determine the prognostic value of the TMEindex based on the clinical characteristics of the patients. There was no significant difference in TMEindex between patients < 15 years and those ≥ 15 years as well as between the genders (Additional file 1: Fig. S7A). Survival analysis showed that OS and RFS between patients with high and low TMEindex in subgroups with different age and gender had significant differences, with shorter OS and RFS for patients in the high TMEindex group (Additional file 1: Fig. S7B–E).

### Validation of the TMEindex in independent cohorts

**Fig. 3:**
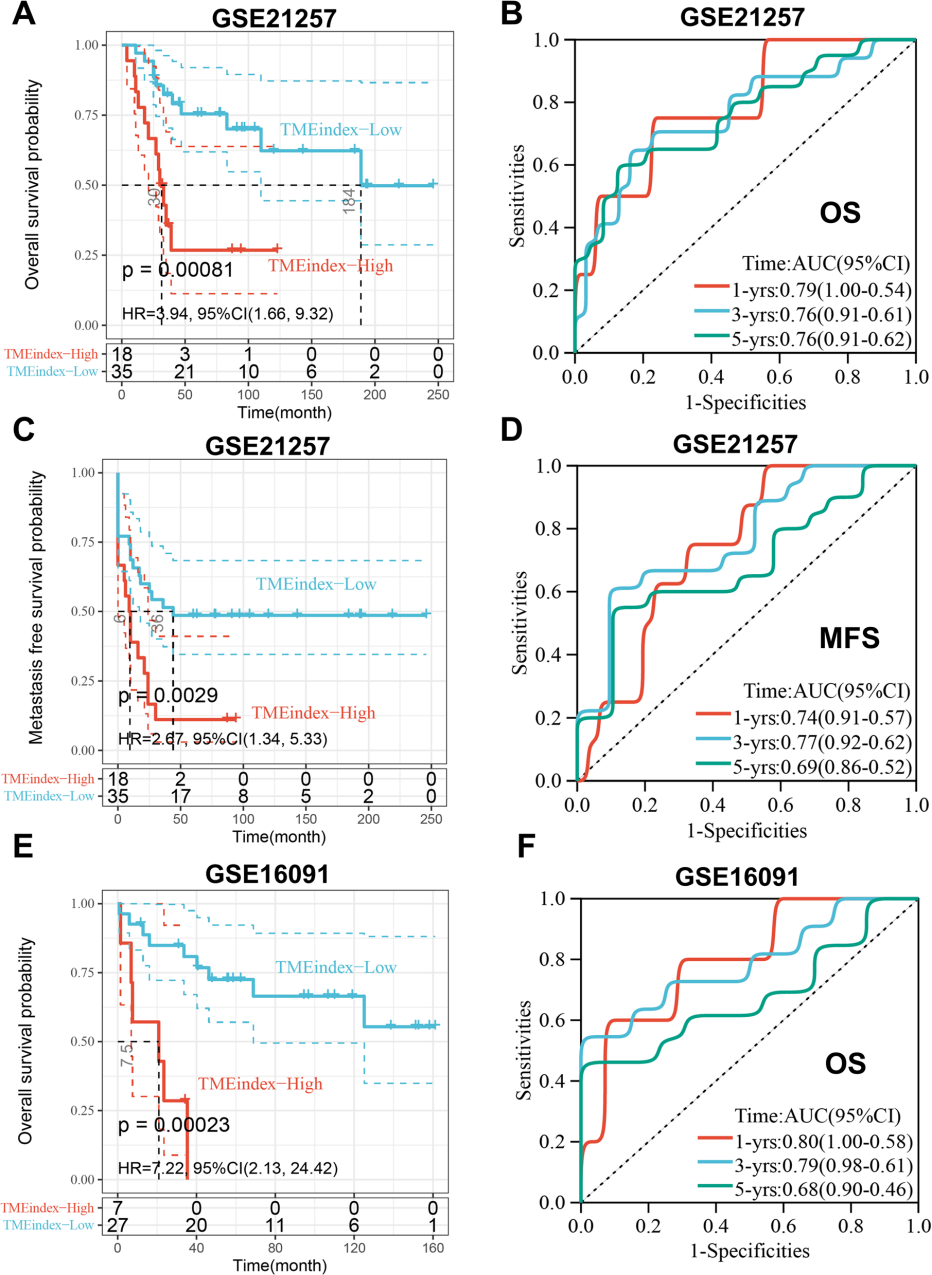
Validation of the prognostic predictive power of TMEindex in independent cohorts.** A** Kaplan–Meier curve depicts the OS difference between TMEindex-high and TMEindex-low groups (log-rank P = 0.00081) in the GSE21257 cohort. **B** ROC curve showing the OS prediction efficiency of the TMEindex in the GSE21257 cohort. **C** Kaplan–Meier curve depicts the MFS difference between TMEindex-high and TMEindex-low groups (log-rank P = 0.0029) in the GSE21257 cohort. **D** ROC curve showing the MFS prediction efficiency of the TMEindex in the GSE21257 cohort. **E** Kaplan–Meier curve depicts the OS difference between TMEindex-high and TMEindex-low groups (log-rank P = 0.00023) in the GSE16091 cohort. **F** ROC curve showing the OS prediction efficiency of the TMEindex in the GSE16091 cohort

To further assess the robustness of the TMEindex, we selected three independent datasets (GSE21257, GSE16091 and GSE33382) to validate the prognostic predictive power of the TMEindex. In the GSE21257 dataset, the KM curve showed that the high TMEindex group had significantly worse OS (*P* = 0.00081; Fig. 3A). The AUCs of 1, 3, and 5-year OS were 0.79, 0.76, 0.76, respectively (Fig. 3B). Additionally, the high TMEindex group also had worse metastasis-free survival (MFS) (*P* = 0.0029; Fig. 3C) and the AUCs of the TMEindex were 0.74, 0.77 and 0.69 at 1, 3, and 5-year of MFS (Fig. 3D). In the GSE21257 cohort we analyzed the two most common subtypes of osteosarcoma separately. The high TMEindex group had worse OS and MFS in both osteoblastic and chondroblastic osteosarcoma (Additional file 1: Fig. S8A, B), although in chondroblastic osteosarcoma there was no statistical significance due to the sample size being too small. In the GSE16091 dataset, patients with high TMEindex were also validated to have worse OS than patients with low TMEindex (*P* = 0.00023; Fig. 3E). The ROC curve showed excellent predictive performance (Fig. 3F). Due to the lack of time data to MFS in the GSE33382 dataset, we explored the prediction power of the TMEindex on metastasis status in this dataset. Patients who did not develop metastases within 5 years had significantly lower TMEindex compared to those who didn’t develop metastases within 5 years (Additional file 1: Fig. S9A). Additionally, increased TMEindex was associated with greater probability of metastasis within 5 years, and the TMEindex showed good predictive performance for metastatic status with AUC of 0.69 (Additional file 1: Fig. S9B).

### TMEindex is an independent risk factor of osteosarcoma

To evaluate whether the prognostic performance of TMEindex in osteosarcoma is independent of other clinical factors, we performed univariate and multivariate Cox analyses (Additional file 1: Table S5). For OS, univariate Cox analysis indicated that TMEindex was associated with unfavorable OS in both the TARGET cohort (*P* < 0.001) and the GSE21257 dataset (*P* = 0.021), while multivariate Cox analysis indicated that TMEindex was an independent risk factor for OS in both cohorts (*P* < 0.001and *P* = 0.013). In the GSE16091 dataset, although the univariate Cox analysis failed to show a significant correlation between TMEindex and OS (*P* = 0.051), TMEindex was significantly associated with unfavorable OS in multivariate Cox analysis after adjusted by age and gender (*P* = 0.049). For RFS, both univariate (*P* < 0.001) and multivariate Cox analyses (*P* < 0.001) in the TARGET cohort suggested that TMEindex was significantly associated with unfavorable RFS in osteosarcoma. For MFS, the univariate (*P* = 0.003) and multivariate (*P* = 0.014) Cox analyses in the GSE21257 dataset also showed that TMEindex was associated with unfavorable MFS.

### The scRNA-seq analysis of TMEindex genes

**Fig. 4:**
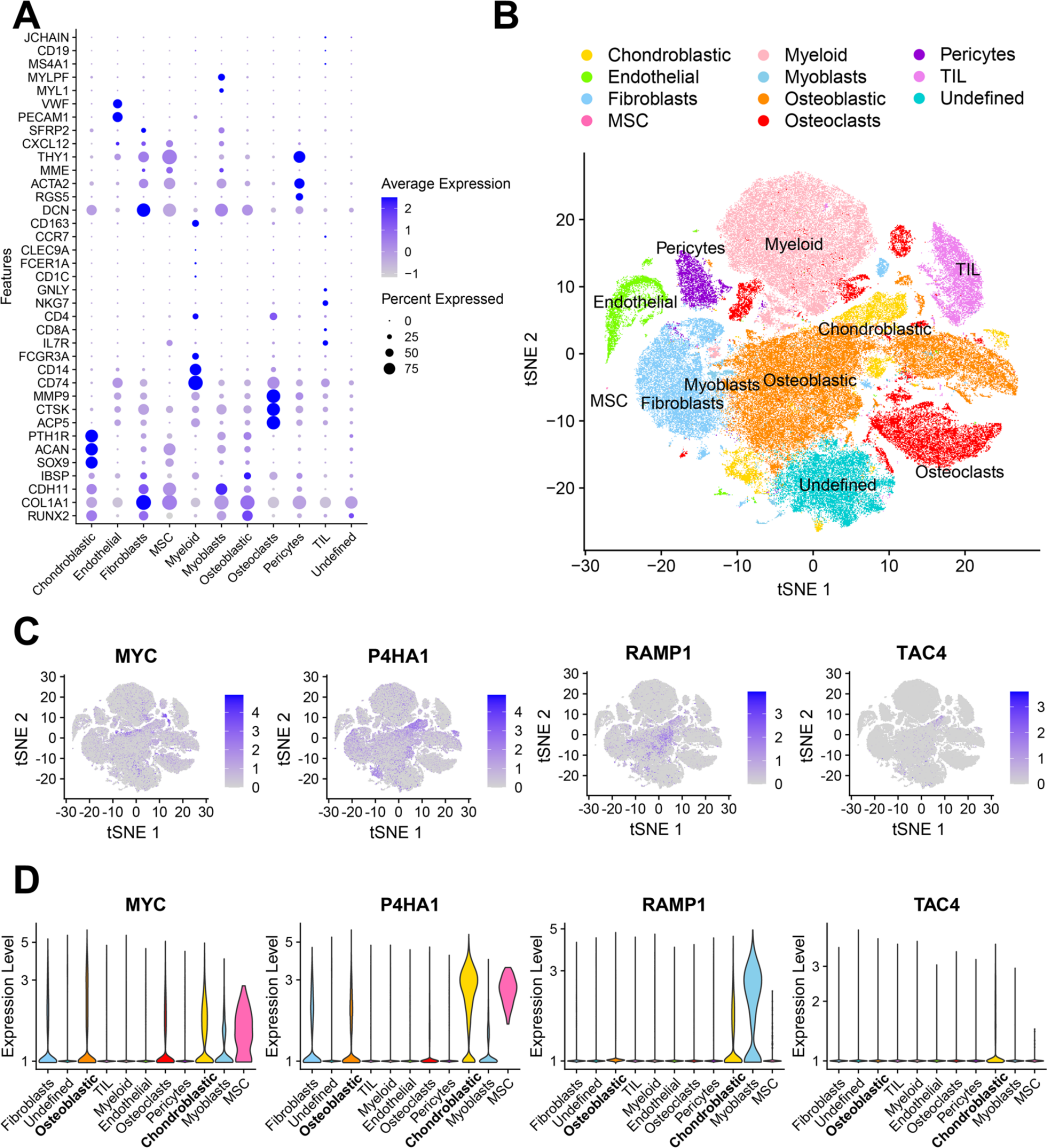
The scRNA-seq analysis of TMEindex genes.** A** The dot plot shows the expression of 37 signature genes in 11 cell clusters. The size of the dots indicates the proportion of cells expressing a specific marker, and the color indicates the average expression level of the markers. **B** The t-SNE plot of the 11 main cell types in osteosarcoma. **C** Feature plots for MYC, P4HA1, RAMP1 and TAC4. The color legend shows the normalized expression levels of the genes. **D** Violin plots showing the normalized expression levels of MYC, P4HA1, RAMP1 and TAC4 across the 11 cell types

To further understand the distribution of TMEindex genes in osteosarcoma TME, we analyzed the scRNA-Seq data from osteosarcoma patients. 11 major clusters were identified in osteosarcoma TME based on the expression of characteristic genes (Fig. 4A). The t-SNE plot demonstrated the 11 annotated cell clusters (Fig. 4B), osteosarcoma TME was mainly composed of osteoblastic and chondroblastic osteosarcoma cells, osteoclasts, fibroblasts, myeloid cells, and tumor-infiltrating lymphocytes (TILs). Myoblasts and mesenchymal stem cells (MSCs) occupied a very low percentage of cells in TME. As shown in t-SNE plots and violin plots (Fig. 4C, D), among the major cell types, MYC was mainly expressed in malignant tumor cells, osteoblasts and fibroblasts. P4HA1 was mainly expressed in malignant tumor cells and fibroblasts. RAMP1 was mainly expressed in malignant tumor cells, especially in chondroblastic osteosarcoma cells. The overall expression of TAC4 was low and mainly expressed in chondroblastic osteosarcoma cells.

### Protein expression of TMEindex genes in osteosarcoma tissues and their effects on proliferation, invasion and migration of osteosarcoma cells

**Fig. 5:**
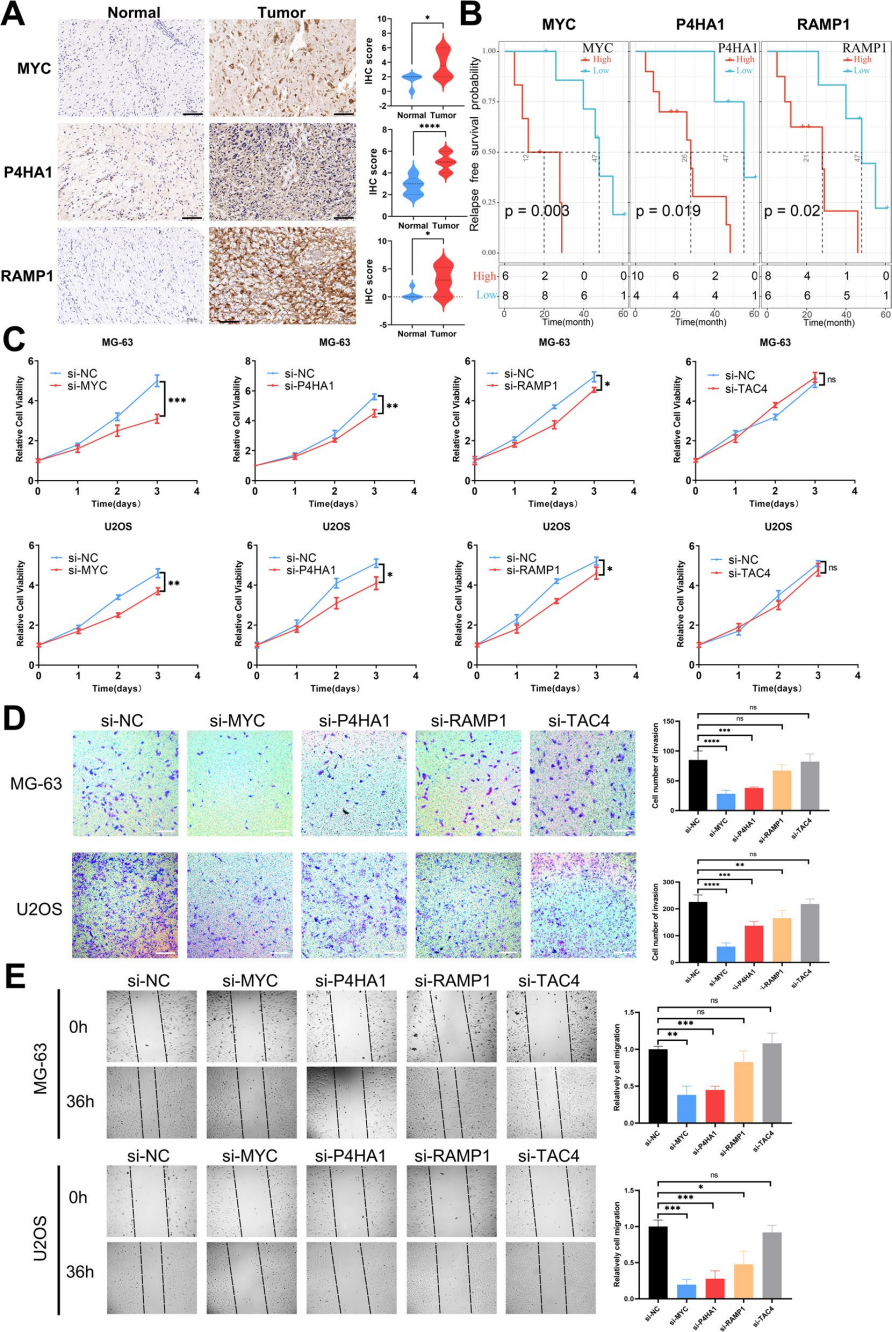
Protein expression of TMEindex genes in osteosarcoma tissues and their effects on proliferation, invasion and migration of osteosarcoma cells.** A** IHC staining images of MYC, P4HA1 and RAMP1 in osteosarcoma tissues and corresponding normal tissues. The IHC scores indicated that the protein expression of MYC, P4HA1 and RAMP1 was higher in tumor tissues. **B** Kaplan–Meier curves depict the RFS difference between high and low TMEindex proteins (MYC, P4HA1 and RAMP1) groups (all log-rank P < 0.0001) in the external cohort. Red representing the high TMEindex proteins group and blue representing the low TMEindex proteins group. **C** Folded line plots showing the effect of MYC, P4HA1, RAMP1 and TAC4 knockdown on the proliferation of MG-63 and U2OS cells. The blue line represents the control group and the red line represents the knockdown group. **D** Transwell chamber experiment showing the effect of MYC, P4HA1, RAMP1 and TAC4 knockdown on the invasion of MG-63 and U2OS cells. Scale bar: 100 μm. **E** Scratch assay showing the effect of MYC, P4HA1, RAMP1 and TAC4 knockdown on the migration of MG-63 and U2OS cells. Data are represented as mean ± SEM. *P < 0.05, **P < 0.01, ***P < 0.001, ****P < 0.0001

To further understand whether the proteins of the TMEindex genes are potential biomarkers, we first performed IHC analysis using osteosarcoma tissues and corresponding normal tissues. Although there were no available antibodies to TAC4, the protein expression of MYC, P4HA1, and RAMP1 was found to be significantly higher in osteosarcoma tissues than in normal tissues (Fig. 5A). In addition, in the independent validation cohort, it was found that osteosarcoma patients with high MYC, P4HA1, and RAMP1 protein expression had worse RFS (Fig. 5B). Further, the effect of TMEindex genes on the malignant phenotype of osteosarcoma cells was explored by molecular biology experiments. As shown in Fig. 5C, cell proliferation was inhibited after knockdown of MYC, P4HA1 and RAMP1 in both MG-63 and U2OS cells, while cell proliferation was not affected after knockdown of TAC4. In addition, the cell invasion and migration abilities of both MG-63 and U2OS were inhibited after knockdown of MYC and P4HA1, and no change in cell invasion and migration ability was observed after knockdown of TAC4 (Fig. 5D, E). Interestingly, the invasive and migratory abilities of U2OS cells were inhibited after knockdown of RAMP1, while the invasive and migratory abilities of MG-63 cells were not.

### Molecular characteristics of the TMEindex

**Fig. 6:**
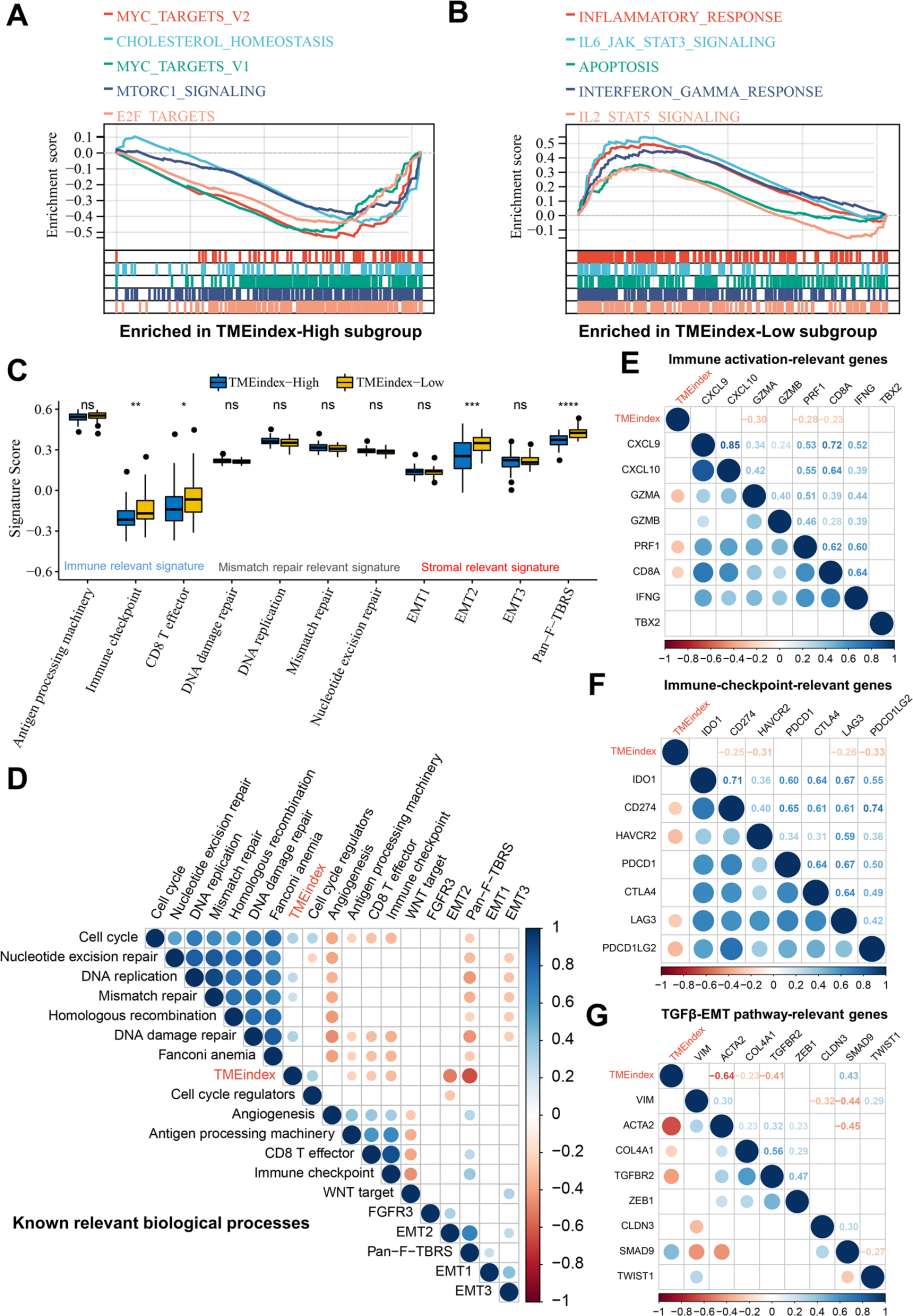
Molecular characteristics of the TMEindex.** A**,** B** GSEA enrichment plots base on HALLMARK gene set showing the relatively enriched pathways in TMEindex-high (**A**) and TMEindex-low (**B**) groups. **C** Differences in different signatures (immune relevant signature, mismatch repair relevant signature, and stromal relevant signature as indicated) between TMEindex-high and TMEindex-low groups. The upper and lower ends of the boxes represented interquartile range of values. The lines in the boxes represented median value, and black dots showed outliers. *P < 0.05, **P < 0.01, ***P < 0.001, ****P < 0.0001. **D** Correlations between TMEindex and known core biological processes signature scores. **E** Correlations between TMEindex and immune activation-relevant genes expression. **F** Correlations between TMEindex and immune-checkpoint-relevant genes expression. **G** Correlations between TMEindex and TGFβ/EMT pathway-relevant genes expression. Correlation coefficients are calculated by Spearman’s correlation analysis, with red representing negative correlations and blue representing positive correlations. Blank represents a correlation P-value > 0.05

To explore the potential mechanisms leading to the different prognostic outcomes between the high and low TMEindex groups, a GSEA based on the HALLMARK gene set was first performed. As shown in Fig. 6A, B, the high TMEindex samples were mainly enriched in MYC target genes, E2F target genes and MTOR-related pathway, while the low TMEindex samples were mainly enriched in apoptosis and immune response-related biological processes such as inflammatory response and interferon gamma (IFNγ) response. To further confirm these results, GSEA was performed again based on the KEGG gene set. Not surprisingly, apoptosis and immune response-related pathways such as antigen processing and presentation, and T/B cell receptor signaling pathways were enriched in the TMEindex low group (Additional file 1: Fig. S10B), while DNA replication, mismatch repair (MMR) and ribosome-related pathways were enriched in the high TMEindex group (Additional file 1: Fig. S10A). Further, a total of 612 DEGs were identified between high and low TMEindex groups (Additional file 2: Table S6; Additional file 1: Fig. S11A). The KEGG pathway analysis of these DEGs revealed that they were mainly involved in PI3K-Akt signaling pathway, Wnt signaling pathway, focal adhesion and other oncogenic related pathways (Additional file 1: Fig. S11B). GO enrichment analysis revealed that DEGs were closely related to immune-related biological processes and extracellular matrix (Additional file 1: Fig. S11C), which is consistent with the previous results.

The previous findings suggested that the TMEindex was indeed closely related to the immune process; therefore, immune-related signatures, stromal-related signatures, and mismatch repair-related signatures were tested in the different TMEindex groups. As shown in Fig. 6C, the low TMEindex group had higher immune-related signature scores (immune checkpoint and CD8 T effector) and stromal-related signature scores (EMT2 and Pan-F-TBRS). To better characterize the function of the TMEindex, the correlation between the TMEindex and known core biological processes was also tested. TMEindex was found to be positively correlated with cell cycle, DNA replication, MMR, and DNA damage repair (DDR), signatures, while negatively correlated with antigen processing, CD8 T effector, immune checkpoint, EMT, and Pan-F-TBRS signatures (Fig. 6D). To characterize the immune-related biological processes of TMEindex, an analysis was performed based on a study by Zeng et al. [29]. Among immune activation-relevant transcripts, TMEindex was found to be positively correlated with GZMA, PRF1 and CD8A (Fig. 6E). Among the immune checkpoint-relevant transcripts, TMEindex was positively correlated with CD274, HAVCR2, LAG3 and PDCD1LG2 (Fig. 6F). TMEindex was also positively associated with ACTA2, COL4A1 and TGFBR2 among the TGFβ/EMT pathway-relevant transcripts (Fig. 6G).

### Immune cell infiltration characteristics of TMEindex

**Fig. 7:**
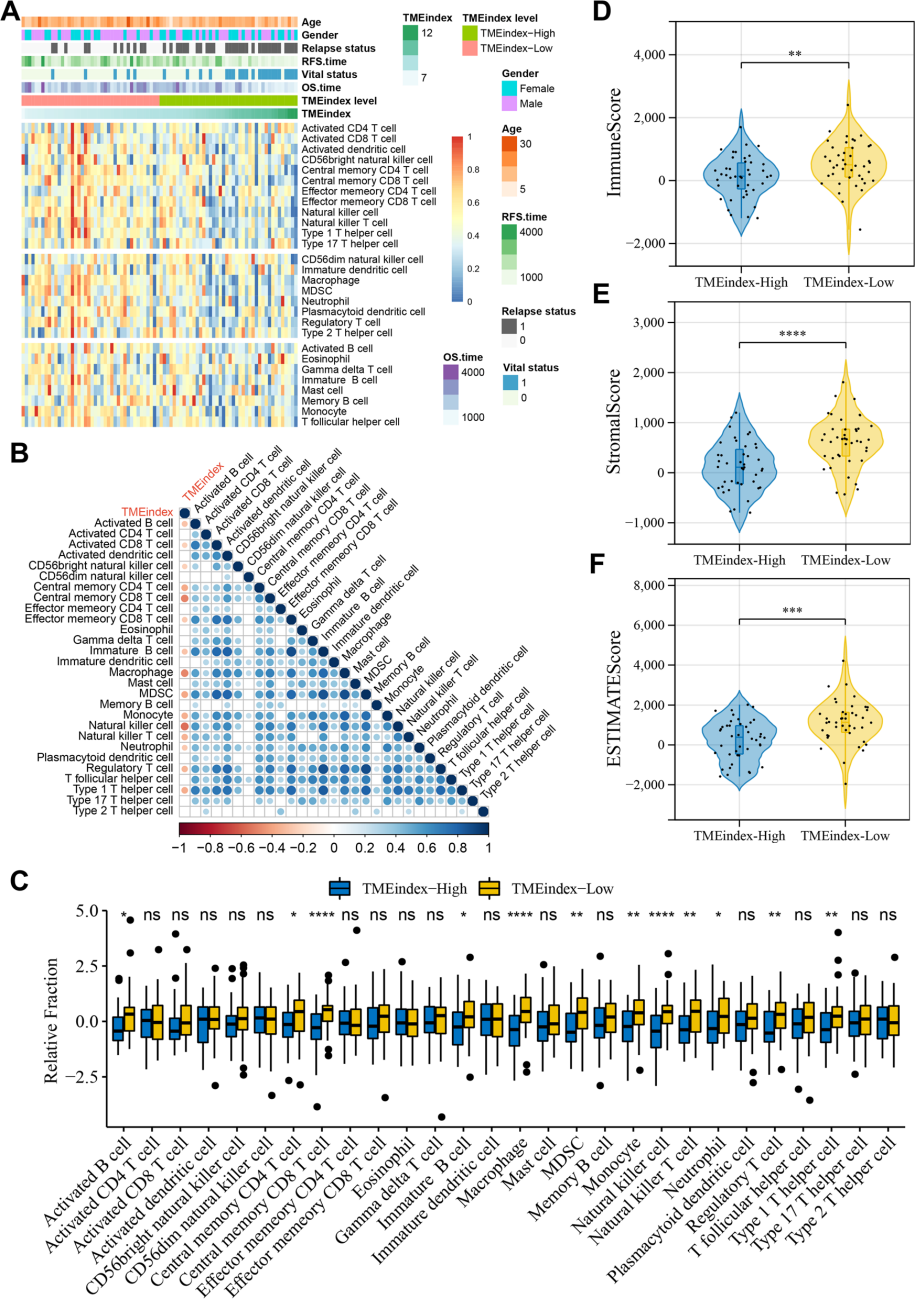
Immune and stromal cell infiltration characteristics of TMEindex.** A** Heatmap of the relationship between TMEindex and 28 immune and stromal cells. Age, gender, vital status, OS time, relapse status and RFS time are shown as patient annotations. **B** Correlations of TMEindex with abundance of 28 immune and stromal cells. Correlation coefficients are calculated by Spearman’s correlation analysis, with red representing negative correlations and blue representing positive correlations. Blank represents a correlation P-value > 0.05. **C** Differences in 28 immune and stromal cells between TMEindex-high and TMEindex-low groups. The upper and lower ends of the boxes represented interquartile range of values. The lines in the boxes represented median value, and black dots showed outliers. **D–F** Differences in ImmuneScore (**D**), StromalScore (**E**) and ESTIMATEScore (**F**) between TMEindex-high and TMEindex-low groups. The blue represents the TMEindex-high group and the yellow represents TMEindex-low group. *P < 0.05, **P < 0.01, ***P < 0.001, ****P < 0.0001

The previous results have identified that TMEindex is closely associated with TME as well as immune response in osteosarcoma, and to further characterize the TMEindex in relation to the immune landscape, ssGSEA was performed to extrapolate the relative abundance of 28 immune cells. Figure 7A illustrated the distribution characteristics of immune cells associated with TMEindex, also included the clinical characteristics of different TMEindex subgroups. Specifically, in the high TMEindex group, activated B cells, central memory CD4 and CD8 T cells, immature B cell, macrophages, myeloid-derived suppressor cells (MDSCs), natural killer (NK) cells, NK T cells, monocytes, neutrophils, regulatory T cells (Tregs) and type 1T helper (Th1) cells infiltration were significantly reduced (Fig. 7C). Correlation analysis revealed that TMEindex was negatively correlated with the infiltration levels of most immune activating cells (e.g. activated CD8 T cells, NK cells and Th1 cells) and immunosuppressive cells (e.g. CD56dim NK cells, MDSCs and Tregs) (Fig. 7B). In addition, the low TMEindex group was found to have significantly higher ImmuneScore (P < 0.01; Fig. 7D), StromalScore (P < 0.0001; Fig. 7E), and ESTIMATEScore (P < 0.001; Fig. 7F) than the high TMEindex group. The above results further confirmed that TMEindex was negatively correlated with the infiltration level of immune cell in the TME of osteosarcoma.

### Pan-cancer analysis of the prognostic predictive value of TMEindex

Based on univariate Cox analysis, we evaluated the prognostic value of the TMEindex in 37 tumor types, including 9798 tumor samples. The results showed that TMEindex was identified as an unfavorable prognostic biomarker for OS in nine tumors (Additional file 1: Fig. S12A), including kidney renal papillary cell carcinoma (KIRP), BLCA, adrenocortical carcinoma (ACC), head and neck squamous cell carcinoma (HNSC), pancreatic adenocarcinoma (PAAD), cervical squamous cell carcinoma and endocervical adenocarcinoma, uveal melanoma (UVM), lung adenocarcinoma and ovarian serous cystadenocarcinoma. Interestingly, the TMEindex was considered to be a favorable prognostic biomarker for OS in lower grade glioma (LGG). Further, TMEindex was also found to be an unfavorable biomarker of disease-free survival (DFS) in KIPR, ACC and PAAD (Additional file 1: Fig. S12B). For progression-free survival (PFS), TMEindex was identified as an unfavorable biomarker in KIRP, PAAD, UVM, ACC, kidney chromophobe, HNSC and BLCA (Additional file 1: Fig. S13). Consistent with in OS, the TMEindex was also identified as a favorable biomarker for PFS in LGG.

### Potential of TMEindex to predict immunotherapy, chemotherapy and targeted therapy response

**Fig. 8:**
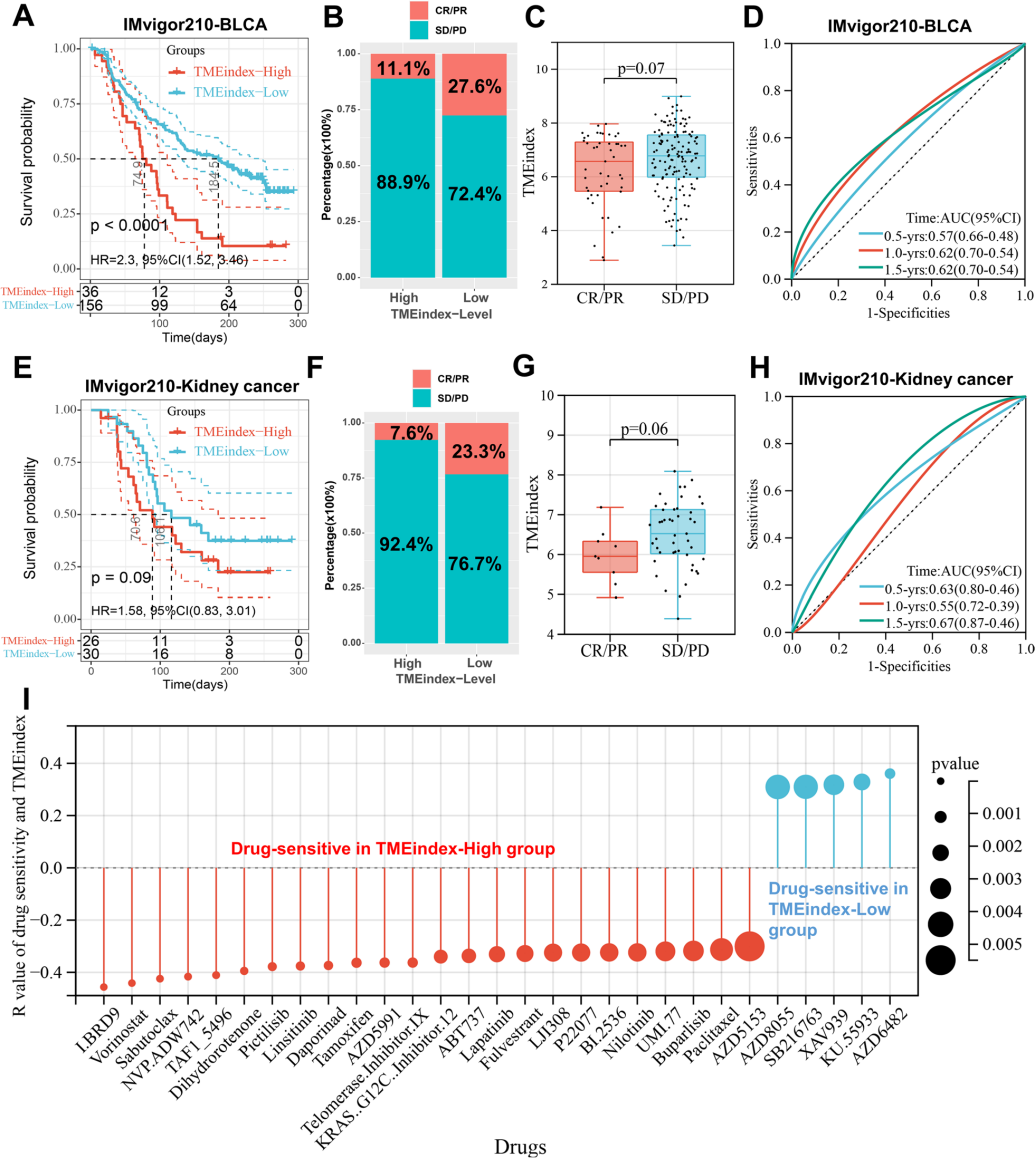
The relationship between TMEindex and efficacy of immunotherapy and drug sensitivity.** A**,** E** Kaplan–Meier curves depict the OS difference between TMEindex-high and TMEindex-low groups after anti-PD-L1 immunotherapy in the IMvigor210-BLCA (**A**, log-rank P < 0.0001) and IMvigor210-Kidney cancer (**B**, log-rank P = 0.09) cohorts. **B**,** F** Rate of clinical response (complete response [CR]/partial response [PR] and stable disease [SD]/progressive disease [PD]) to anti–PD-L1 immunotherapy in TMEindex-high and TMEindex-low groups in the IMvigor210-BLCA (**B**) and IMvigor210-Kidney cancer (**F**) cohorts. **C, G** TMEindex in groups with different anti–PD-L1 clinical response status in the IMvigor210-BLCA (**C**) and IMvigor210-Kidney cancer (**G**) cohorts. The red represents CR/PR patients and the blue represents SD/PD patients. **D, H** ROC curves showing the OS prediction efficiency of the TMEindex in the IMvigor210-BLCA (**D**) and IMvigor210-Kidney cancer (**H**) cohorts. **I** The correlation between TMEindex and drug sensitivity (IC50 value). Each column represents a drug. The height of the column represents the correlation coefficient. The red represents drugs sensitive in TMEindex-high group and the blue represents drugs sensitive in TMEindex-low group

There is no doubt that immunotherapy, represented by immune checkpoint inhibitors (ICIs), has emerged a major breakthrough in tumor therapy [35–37]. Next, the prognostic value of TMEindex for the ICI therapy was explored through two cohorts (IMvigor210-BLCA and IMvigor210-Kidney cancer) receiving anti-PD-L1 therapy. In the IMvigor210-BLCA cohort, patients with low TMEindex exhibited significant therapeutic advantages to anti-PD-L1 therapy and a markedly prolonged OS (*P* < 0.001; Fig. 8A). The TMEindex-Low group had a higher proportion of complete response (CR)/partial response (PR) patients compared to the TMEindex-High group (Fig. 8B). Although not significant, CR/PR patients also had a relatively lower TMEindex than stable disease (SD)/progressive disease (PD) patients (P = 0.07; Fig. 8C). The ROC curve confirmed the predictive role of TMEindex on the survival benefit of anti-PD-L1 therapy in BLCA patients (Fig. 8D). Consistent with the IMvigor210-BLCA cohort, patients with high TMEindex in the IMvigor210-Kidney cancer cohort also had relatively poorer OS (P = 0.09; Fig. 8E). In addition, the predictive value of the TMEindex to ICI response was also verified in this cohort (Fig. 8F, G). The ROC curve also demonstrated the predictive effect of the TMEindex on the survival benefit of ICI therapy in kidney cancer patients (Fig. 8H).

To explore the predictive potential of the TMEindex for drug treatment response, we extrapolated the IC50 values of 189 compounds in the TARGET cohort. Significant correlations between the TMEindex and the sensitivity of 29 drugs were determined (Additional file 2: Table S7). As shown in Fig. 8I, patients in the TMEindex-high group were more sensitive to 24 drugs including the cell cycle inhibitor BI.2536 and the Mitosis inhibitor Paclitaxel. Patients in the TMEindex-low group were more sensitive to only five drugs, including Wnt signaling pathway inhibitors SB216763 and XAV939 (Fig. 8I). In addition, the targets of these 29 drugs were analyzed, drugs sensitive in the TMEindex-high group mainly targeted apoptosis regulation, chromatin and genomic integrity, etc., while drugs sensitive in the TMEindex-low group targeted PI3K/mTOR and Wnt signaling pathways as described above (Additional file 2: Table S8).

## Discussion

In this study, we collected osteosarcoma datasets from multiple platforms to develop and validate an osteosarcoma TME-based risk model (TMEindex). We confirmed in several independent datasets that TMEindex is an independent prognostic factor with good predictive power for OS, RFS and MFS. In particular, the ROC curves demonstrated the markedly accuracy of the TMEindex in predicting the prognosis of patients with osteosarcoma. Additionally, stratification analysis further verified the robustness of TMEindex in prognosis prediction. These results from multiple independent datasets support TMEindex as a valid risk stratification model. It is worth noting that TMEindex is a model established based on the osteosarcoma cohort, so its application value in pan-cancer remains to be further validated. In particular, TMEindex is a favorable prognostic marker in LGG. This may be due to the fact that immunosuppressive factors in the TME of glioma have a very critical impact on prognosis [38–42], and TMEindex is negatively correlated with the infiltration of immunosuppression-related cells in tumors, but the reasons need to be further revealed in LGG.

In addition to its prognostic role, this study validated the potential of TMEindex as a quantitative molecular signature of TME in osteosarcoma from multiple perspectives. The TMEindex was negatively correlated not only with multiple immune-related signatures but also with the abundance of multiple immune cells in osteosarcoma TME. Previous studies have typically used the ESTIMATE algorithm to estimate TME for a variety of tumors [23, 43, 44], and Zeng et al. also constructed a model (TMEscore) to quantify TME infiltration in gastric cancer. However, both the ESTIMATE algorithm and the TMEscore involve hundreds and thousands of genes, which means that it is expensive to quantify TME based on them. In the presented study, TMEindex consisted of only four genes, MYC, P4HA1, RAMP1, and TAC4. MYC is a recognized proto-oncogene that plays an important role in cell cycle, proliferation, differentiation, and even global gene expression [45]. Casey et al. found that downregulation of MYC enhanced antitumor responses by regulating CD47 and PD-L1 expression [46]. In osteosarcoma, MYC promotes cell invasion by activating the MEK-ERK pathway and facilitates malignant progression of the tumor [47, 48]. A recent study also shown that MYC is negatively associated with multiple immune cells and immune function in osteosarcoma, which supports the results of our study [49]. P4HA1 is a key enzyme in the synthesis of collagen, which is an important component of TME and is involved in the regulation of tumor immunity [50]. A pan-cancer study showed that P4HA1 is not only associated with poor prognosis in most tumors, but its overexpression also predicts an immunosuppressive TME [51]. P4HA1 has also been reported to be closely associated with the regulation of hypoxic microenvironment and immune infiltration in osteosarcoma [52]. As for RAMP1, this gene is a member of the receptor activity modifying proteins (RAMP) family, is involved in the terminal glycosylation, maturation and presentation of calcitonin gene-related peptide (CGRP) receptors to the cell surface [53]. There is experimental evidence indicating that the expression of RAMP1 in immune cells, including T cells, is critical for inflammation suppression [54, 55] and inflammation-associated lymphangiogenesis [56]. Additionally, targeting the CALCB/RAMP1 axis successfully inhibited tumor growth in a study of Ewing sarcoma [57]. As for TAC4, a member of the tachykinin family that activates neurons and elicits behavioral responses, previous studies have reported its regulatory role in the immune system and inflammatory responses [58]. Tachykinins have been found to promote the production of memory Th17 cells by the inducting the expression of cytokines such as IL-1β in monocytes, thereby modulating the immune response [59]. These studies suggest that the genes involved in the TMEindex represent the direction of regulation of TME and support the TMEindex as a biomarker associated with immune response and tumor malignancy progression. Notably, the TMEindex genes were mainly expressed more highly in malignant cells, which may be a potential reason why patients with higher TMEindex have lower immune and stromal cell infiltration. In addition, although MYC and P4HA1 expression in MSCs and RAMP1 expression in Myoblasts were also higher, they cannot have a significant impact on TMEindex since MSCs and Myoblasts occupied a very low percentage of TME. Further molecular biology experiments showed that MYC and P4HA1 had a significant effect on the malignant phenotype of osteosarcoma cells, while TAC4 had no effect. The effect of RAMP1 on the malignant phenotype of osteosarcoma cells was cell type dependent.

To better characterize and understand the underlying mechanisms of different prognosis between patients with a different TMEindex, we performed a GSEA for the high and low TMEindex groups. The high-TMEindex group had higher enrichment of MYC-related signaling pathways and mTOR signaling pathway. As previously described, MYC promoted tumor proliferation and suppressed anti-tumor responses [46, 47], while aberrant activation of mTOR was a key factor in tumor growth and metastasis [60]. In addition, there was significantly lower anti-tumor lymphocyte infiltration in the high-TMEindex group. In contrast, the low-TMEindex group was predominantly enriched for anti-tumor immune response processes such as inflammatory response and IFNγ response. In addition, the low-TMEindex group had a higher infiltration of immune cells. The current view is that pre-existing antitumor immune responses usually improve the prognosis of cancer patients [61], so the better prognosis in the low-TMEindex group may result from better immune control. Overall, the prognostic value of TMEindex stems from a better immune response and lower tumor malignancy.

With the success of immunotherapy, especially ICI therapy, in a variety of tumors [36, 62, 63], the use of immunotherapy to improve survival outcomes in osteosarcoma has become an attractive strategy. The benefits of immunotherapy strategies based on immunostimulants and innate immune cells have been well documented in early studies [64–66], suggesting promising applications for immunotherapy in osteosarcoma. In addition, the high presence of tumor-infiltrating lymphocytes in osteosarcoma TME and the correlation between CD8 T cells and higher survival rate has led to the hypothesis that ICI therapy could be effective in osteosarcoma [67–69]. Unfortunately, only a very small number of patients in two studies of anti-PD-1 monotherapy for osteosarcoma showed PR [70, 71]. However, the combination of anti-PD-L1 and anti-CTLA-4 therapy showed objective responses and clinical benefits on PFS in metastatic osteosarcoma [72, 73]. Additionally, biological experiments based on a mouse model of osteosarcoma demonstrated the effective inhibition to lung metastasis by PD-1 inhibitors [74]. These encouraging findings provide a potential rationale for further clinical trials. A large number of clinical trials are currently underway with strategies using ICIs combination therapy or ICI in combination with chemotherapy or targeted therapy that are designed to improve the response to ICIs [75, 76]. The foreseeable development of immunotherapy for osteosarcoma highlights the need to identify prognostic biomarkers for immunotherapy of this disease. Tumor mutation burden (TMB) and microsatellite Instability (MSI) tests are genomic biomarkers currently used to identify patients who may benefit from ICIs [77, 78]. Gounder et al. performed targeted panel sequencing on 7494 sarcoma (including osteosarcoma) samples and found that only 3.9% of sarcoma patients had a relevant TMB and the frequency of MSI was extremely low (< 0.3%) [79]. The proportion of relevant TMB and MSI in osteosarcoma was even lower. This suggests that TMB and MSI may not be suitable as valid biomarkers for ICI therapy in osteosarcoma and that new biomarkers need to be identified. Previous studies have shown that the clinical benefit and prognostic outcome of immunotherapy is largely determined by the patient’s TME status [80, 81]. In this study, the TMEindex is a quantitative molecular signature of TME in patients with osteosarcoma. Patients with lower TMEindex have higher ImmuneScores, immune cell infiltration levels and immune activation-related signature scores, which represent higher immune activity. Therefore, patients with a lower TMEindex are more suitable for treatment with ICI to offset the immunosuppression and enhance the existing anti-tumor immunity. As mentioned earlier, ICIs combination therapy is likely to be more effective than ICI monotherapy. Patients with higher PD-L1 expression tended to respond better to anti-PD-1/PD-L1 therapy [82], consistent with the fact that patients with lower TMEindex also had higher expression of checkpoint genes, including PD-L1 (CD274), which further supports our conclusion. On the other hand, patients with higher TMEindex have lower immune cell infiltration and cold TME, therefore patients with high TMEindex may be suitable for tumor vaccines and immunostimulants to enhance anti-tumor immune cell infiltration [83]. Due to the lack of data on osteosarcoma immunotherapy, we were unable to validate it in further clinical trial data. Considering that in the pan-cancer analysis, we found the most significant prognostic predictive effect of TMEindex in KIRC and BLCA, therefore we selected two anti-PD-L1 cohorts in kidney cancer and BLCA for validation. Not surprisingly, kidney cancer and BLCA patients with lower TMEindex were more likely to respond to anti-PD-L1 therapy and achieve longer survival benefits. Although we were unable to find an osteosarcoma ICI treatment cohort for validation, this still provides a further theoretical basis for the TMEindex to predict ICI treatment response in osteosarcoma. In addition, this also provides a reference for the future application of TMEindex in other tumors.

In addition to immunotherapy, the feasibility of TMEindex as a sensitivity marker for chemotherapy and targeted therapy was also investigated in this study. A large number of new targeted drugs for osteosarcoma are currently in development [4]. The TMEindex correlated positively with the sensitivity of 24 drugs targeting multiple pathways, meaning that patients with higher TMEindex were more likely to benefit from these drugs. This included three drugs (Buparlisib, LJI308, and Pictilisib) targeting the PI3K/mTOR pathway, which has been identified as a potential drug target in osteosarcoma [4, 84], and consistent with this, patients with high TMEindex in this study did have higher activation of this pathway. Moreover, these drugs also included two drugs (Linsitinib and NVP.ADW742) that target the insulin-like growth factor pathway (IGF1R), the anti-IGF1R strategy has achieved great success in previous clinical trials [4, 85, 86]. A variety of other drugs, including cell cycle pathway inhibitor BI.2536, are also promising new therapies [4]. Not to be overlooked, two (SB216763 and XAV939) of five drugs whose sensitivity was negatively correlated with TMEindex were targeting the Wnt pathway. A recent study suggests that the lack of response to anti-PD-1 therapy in osteosarcoma patients may be due to an increase in Wnt signaling in immunosuppressive TME [87]. This study has demonstrated that patients with low TMEindex may be more likely to respond to ICI and that low TMEindex is likewise more sensitive to Wnt pathway inhibitors. Therefore, the use of Wnt pathway inhibitors at low TMEindex to improve TME and enhance the efficacy of ICI therapy may be a promising strategy. In conjunction with the previous description, we further recommend trying ICI in combination with chemotherapy or targeted therapy (especially Wnt pathway inhibitors) in low-TMEindex patients and trying multiple chemotherapy or targeted therapy in combination with immunostimulants in high-TMEindex patients.

Some limitations of our findings remain. We were unable to analyze the association between TMEindex and TMB and MSI to provide more information, because mutation data of osteosarcoma patients in the TARGET database was not available. Also, we were unable to find the ICI treatment data for osteosarcoma to validate the predictive effect of TMEindex on the response to ICI treatment for osteosarcoma. The predictive role of the TMEindex on prognosis and immunotherapy response needs to be validated in further prospective cohorts. Finally, additional in vivo and in vitro experiments are needed to explore the effect of the TMEindex genes on drug sensitivity. Future work should reveal the biological mechanisms of TMEindex and incorporate more clinical factors to improve accuracy.

## Conclusions

In conclusion, we developed a promising predictive model based on the osteosarcoma TME. The TMEindex can be used to distinguish molecular and immunological characteristics, predict prognosis and provide a potential reference for the clinical benefit of ICI therapy in osteosarcoma.

## Supplementary Information

**Additional file 1: Table S1**. Clinical characteristics of patients with osteosarcoma in each dataset. **Table S5**. Univariate and multivariate Cox regression analysis of overall survival, relapse free survival and metastasis free survival in each data set. **Figure S1**. The flow diagram of this study. **Figure S2**. ImmuneScore and StromalScore associate with clinical features and outcomes. **Figure S3**. Analysis of network topology for various soft-thresholding powers. **Figure S4**. OS prediction value of 4 genes in the TMEindex. **Figure S5**. RFS prediction value of 4 genes in the TMEindex. **Figure S6**. Differences in survival time and TMEindex genes expression between high and low TMEindex groups. **Figure S7**. Stratified analysis to further determine the prognostic value of the TMEindex based on the clinical characteristics of patients. **Figure S8**. Progonsis prediction value of the TMEindex in osteoblastic and chondroblastic osteosarcoma in the GSE21257 cohort. **Figure S9**. Validation of the prognostic value of the TMEindex in GSE33382. **Figure S10**. GSEA enrichment plots base on KEGG gene set showing the relatively enriched pathways in TMEindex-high (A) and TMEindex-low (B) group. **Figure S11**. GO enrichment and KEGG pathway analysis of DEGs identified between high and low TMEindex groups. **Figure S12**. Pan-cancer analysis of the prognostic predictive value of TMEindex. **Figure S13**. Univariate Cox regression analysis reveals the association of TMEindex with the PFS of 32 tumor types.

**Additional file 2**: **Table S2**. DEGs between high- and low-ImmuneScore groups. **Table S3**. DEGs between high- and low-StromalScore groups. **Table S4**. Genes of green, blue and yellow modules in WGCNA. **Table S6**. DEGs between high- and low-TMEindex groups. **Table S7**. Correlations between TMEindex and IC50 value in different drugs. **Table S8**. Target pathway of 29 TMEindex-related drugs.

## Data Availability

The data generated in this study are available within the article and its supplementary data files. The data analyzed in this study were obtained from Xena Functional Genomics Explorer (https://xenabrowser.net/datapages/), IMvigor210 Core Biologies (http://research-pub.gene.com/IMvigor210CoreBiologies) and the GEO database (https://www.ncbi.nlm.nih.gov/geo/).

## Abbreviations

TME: Tumor microenvironment
ICI: Immune checkpoint inhibitor
TARGET: Therapeutically applicable research to generate effective treatments
GEO: Gene Expression Omnibus
TCGA: The Cancer Genome Atlas
BLCA: Bladder urothelial carcinoma
DEG: Differentially expressed gene
FDR: False discovery rate
FC: Fold change
WGCNA: Weighted gene co-expression network analysis
LASSO: Least absolute shrinkage and selection operator
GSEA: Gene set enrichment analysis
GO: Gene Ontology
KEGG: Kyoto Encyclopedia of Genes and Genomes
Pan-F-TBRS: Pan-fibroblast TGFβ response signature
TGF: Transforming growth factor
EMT: Epithelial–mesenchymal transition
ssGSEA: Single-sample gene-set enrichment analysis
GDSC: Genomics of Drug Sensitivity in Cancer
IC50: Half-maximal inhibitory concentration
KM: Kaplan–Meier
HR: Hazard ratios
ROC: Receiver operating characteristic
AUC: Area under the curve
OS: Overall survival
RFS: Relapse-free survival
MFS: Metastasis-free survival
DFS: Disease-free survival
PFS: Progression-free survival
IFNγ: Interferon gamma
MMR: Mismatch repair
DDR: DNA damage repair
MDSC: Myeloid-derived suppressor cell
NK: Natural killer
Treg: Regulatory T cells
Th1: Type 1T helper
KIRP: Kidney renal papillary cell carcinoma
ACC: Adrenocortical carcinoma
HNSC: Head and neck squamous cell carcinoma
PAAD: Pancreatic adenocarcinoma
UVM: Uveal melanoma
LGG: Lower grade glioma
CR: Complete response
PR: Partial response
SD: Stable disease
PD: Progressive disease
RAMP: Receptor activity modifying proteins
TMB: Tumor mutational burden
MSI: Microsatellite instability
scRNA-seq: Single-cell RNA-sequencing
siRNA: Small interfering RNA
IHC: Immunohistochemistry
TIL: Tumor-infiltrating lymphocyte
MSC: Myoblasts and mesenchymal stem cell
