# Supplementary material for: A tumor microenvironment-based prognostic index for osteosarcoma
Source: J Biomed Sci. 2023 Apr 13;30:23. doi: 10.1186/s12929-023-00917-3 (PMC10099847; doi:10.1186/s12929-023-00917-3)
Supplement: Supplementary file 1 — Additional file 1: Table S1. Clinical characteristics of patients with osteosarcoma in each dataset. Table S5. Univariate and multivariate Cox regression analysis of overall survival, relapse free survival and metastasis free survival in each data set. Figure S1. The flow diagram of this study. Figure S2. ImmuneScore and StromalScore associate with clinical features and outcomes. Figure S3. Analysis of network topology for various soft-thresholding powers. Figure S4. OS prediction value of 4 genes in the TMEindex. Figure S5. RFS prediction value of 4 genes in the TMEindex. Figure S6. Differences in survival time and TMEindex genes expression between high and low TMEindex groups. Figure S7. Stratified analysis to further determine the prognostic value of the TMEindex based on the clinical characteristics of patients. Figure S8. Progonsis prediction value of the TMEindex in osteoblastic and chondroblastic osteosarcoma in the GSE21257 cohort. Figure S9. Validation of the prognostic value of the TMEindex in GSE33382. Figure S10. GSEA enrichment plots base on KEGG gene set showing the relatively enriched pathways in TMEindex-high (A) and TMEindex-low (B) group. Figure S11. GO enrichment and KEGG pathway analysis of DEGs identified between high and low TMEindex groups. Figure S12. Pan-cancer analysis of the prognostic predictive value of TMEindex. Figure S13. Univariate Cox regression analysis reveals the association of TMEindex with the PFS of 32 tumor types. [file 12929_2023_917_MOESM1_ESM.pdf]

Table S1. Clinical characteristics of patients with osteosarcoma in each dataset.

**Characteristics**	**TARGET**	**GSE21257**	**GSE16091**	**GSE33382**
**Platform**	IlluminaHiSeq	IlluminaHiSeq	HG-U133A	IlluminaHiSeq
**Patients, n**	84	53	34	53
**Age**				
<15 years	46	19	23	19
≥15 years	38	34	11	34
**Gender**				
Male	47	34	17	33
Female	37	19	13	20
NA	-	-	4	-
**Vital status**				
Alive	57	30	19	-
Dead	27	23	15	-
**Relapse status**				
Non-relapse	43	-	-	-
Relapse	41	-	-	-
**Metastasis status**				
Non-metastasis	-	19	-	19
Metastasis	-	34	-	34

Table S5. Univariate and multivariate Cox regression analysis of overall survival, relapse free survival and metastasis free survival in each data set.

Characteristics	Univariate analysis			Multivariate analysis		
	HR	95%CI	*P*-value	HR	95%CI	*P*-value
**Overall survival**						
**TARGET**						
Age	1.001	0.921~1.089	0.976	1.045	0.956~1.144	0.330
Gender (Male/ Female)	0.714	0.335~1.521	0.382	0.524	0.238~1.154	0.108
TMEindex	2.718	2.005~3.684	<0.001	2.879	2.089~3.968	<0.001
**GSE21257**						
Age	1.009	0.975~1.044	0.603	1.031	0.991~1.071	0.122
Gender (Male/ Female)	1.403	0.587~3.348	0.445	1.169	0.463~2.949	0.741
TMEindex	1.687	1.078~2.642	0.021	1.882	1.138~3.110	0.013
**GSE16091**						
Age	1.025	0.957~1.097	0.484	1.017	0.946~1.093	0.637
Gender (Male/ Female)	0.658	0.230~1.886	0.437	0.569	0.192~1.687	0.309
TMEindex	1.599	0.997~2.618	0.051	1.784	1.058~2.997	0.049
**Relapse free survival**						
**TARGET**						
Age	0.953	0.887~1.025	0.198	0.982	0.915~1.055	0.622
Gender (Male/ Female)	0.971	0.524~1.802	0.927	1.036	0.547~1.963	0.913
TMEindex	2.146	1.680~2.740	<0.001	2.120	1.653~2.718	<0.001
**Metastasis free survival**						
**GSE21257**						
Age	0.989	0.958~1.020	0.489	1.003	0.969~1.038	0.852
Gender (Male/ Female)	2.179	1.013~4.685	0.046	1.797	0.819~3.940	0.143
TMEindex	1.672	1.183~2.364	0.003	1.595	1.096~2.322	0.014

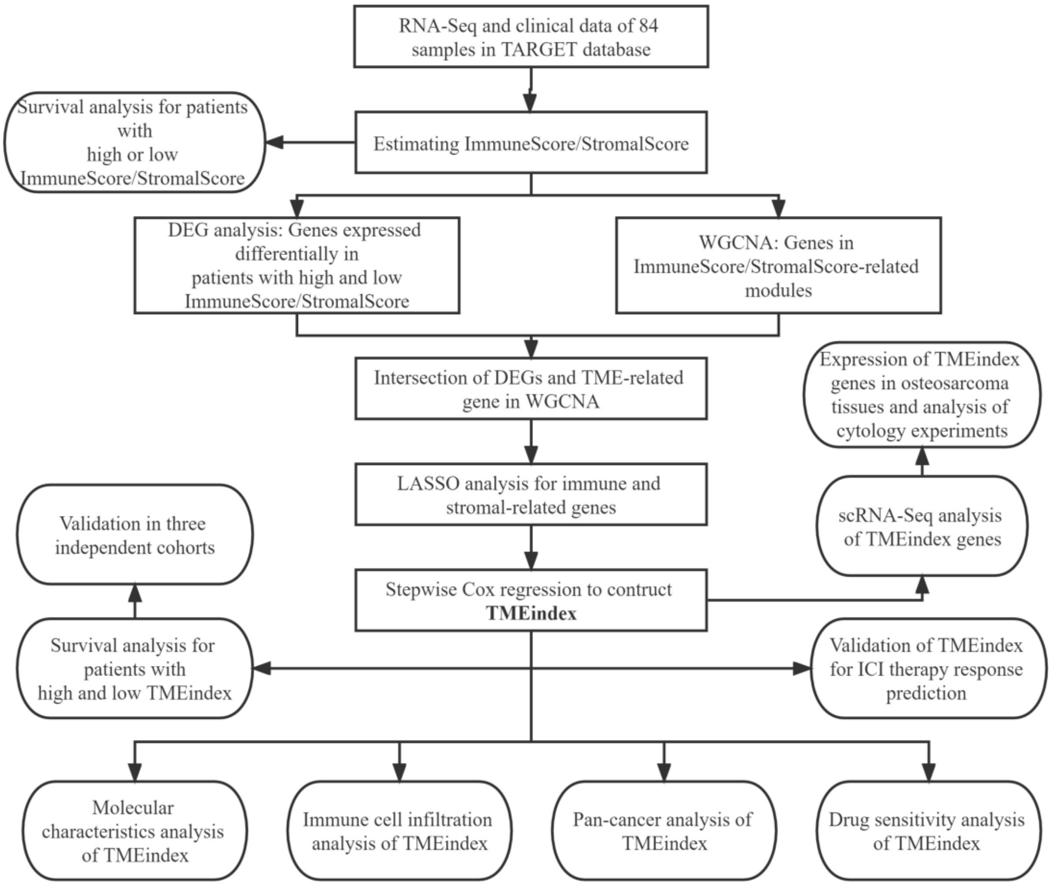

Figure S1. The flow diagram of this study.

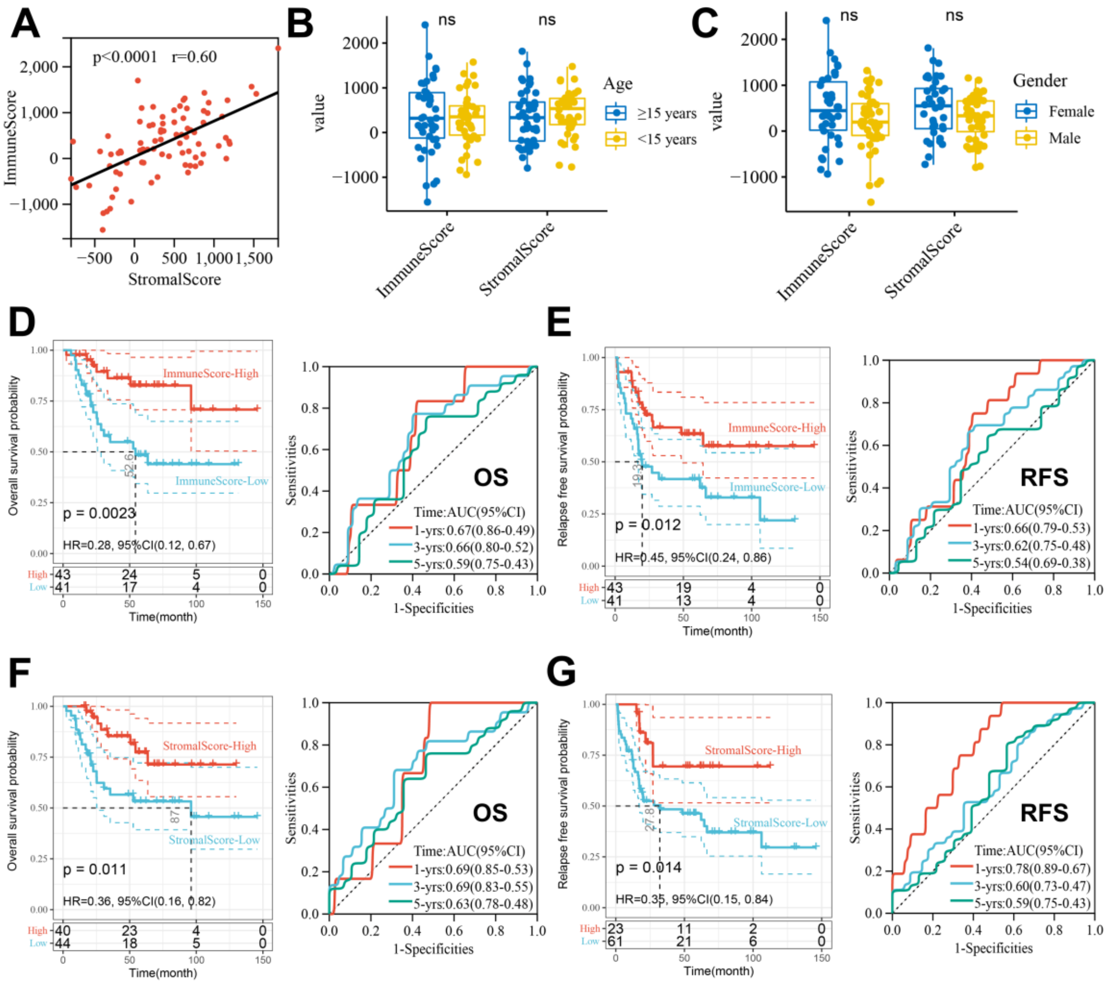

Figure S2. ImmuneScore and StromalScore associate with clinical features and outcomes. A The correlation between ImmuneScore and StromalScore. **B** Differences in ImmuneScore and StromalScore in different age subgroups. The blue represents age ≥ 15 years group and the yellow represents age < 15 years group. **C** Differences in ImmuneScore and StromalScore in different gender subgroups. The blue represents female group and the yellow represents male group. **D,E** Kaplan-Meier curve depicting the OS (**D**) and RFS (**E**) difference between ImmuneScore-high and ImmuneScore-low groups and ROC curve showing the OS (**D**) and RFS (**E**) prediction efficiency of ImmuneScore in the TARGET cohort. **F,G** Kaplan-Meier curve depicting the OS (**F**) and RFS (**G**) difference between StromalScore-high and StromalScore-low groups and ROC curve showing the OS (**F**) and RFS (**G**) prediction efficiency of StromalScore in the TARGET cohort.

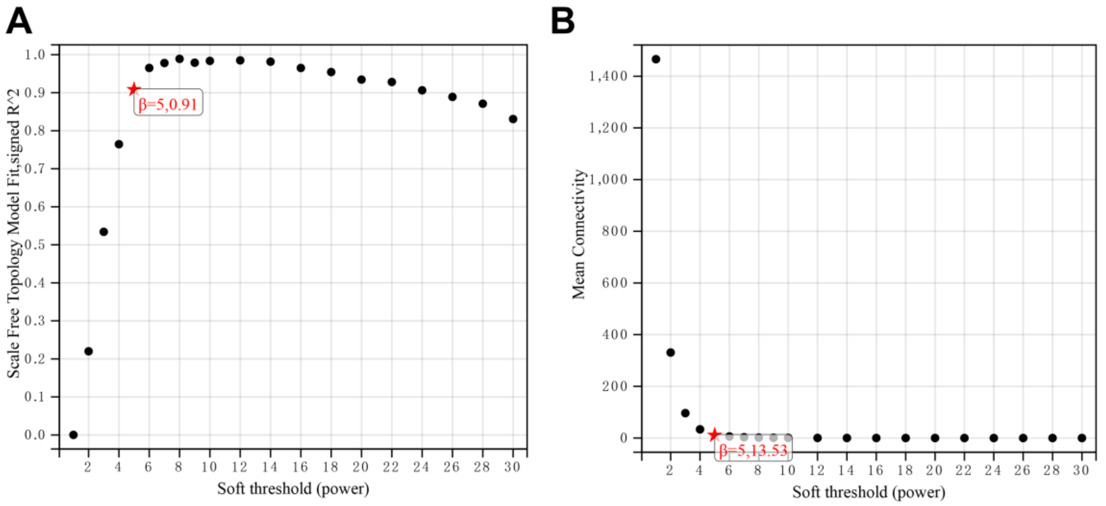

Figure S3. Analysis of network topology for various soft-thresholding powers. A Scale-free network map. **B** Mean connectivity network map.

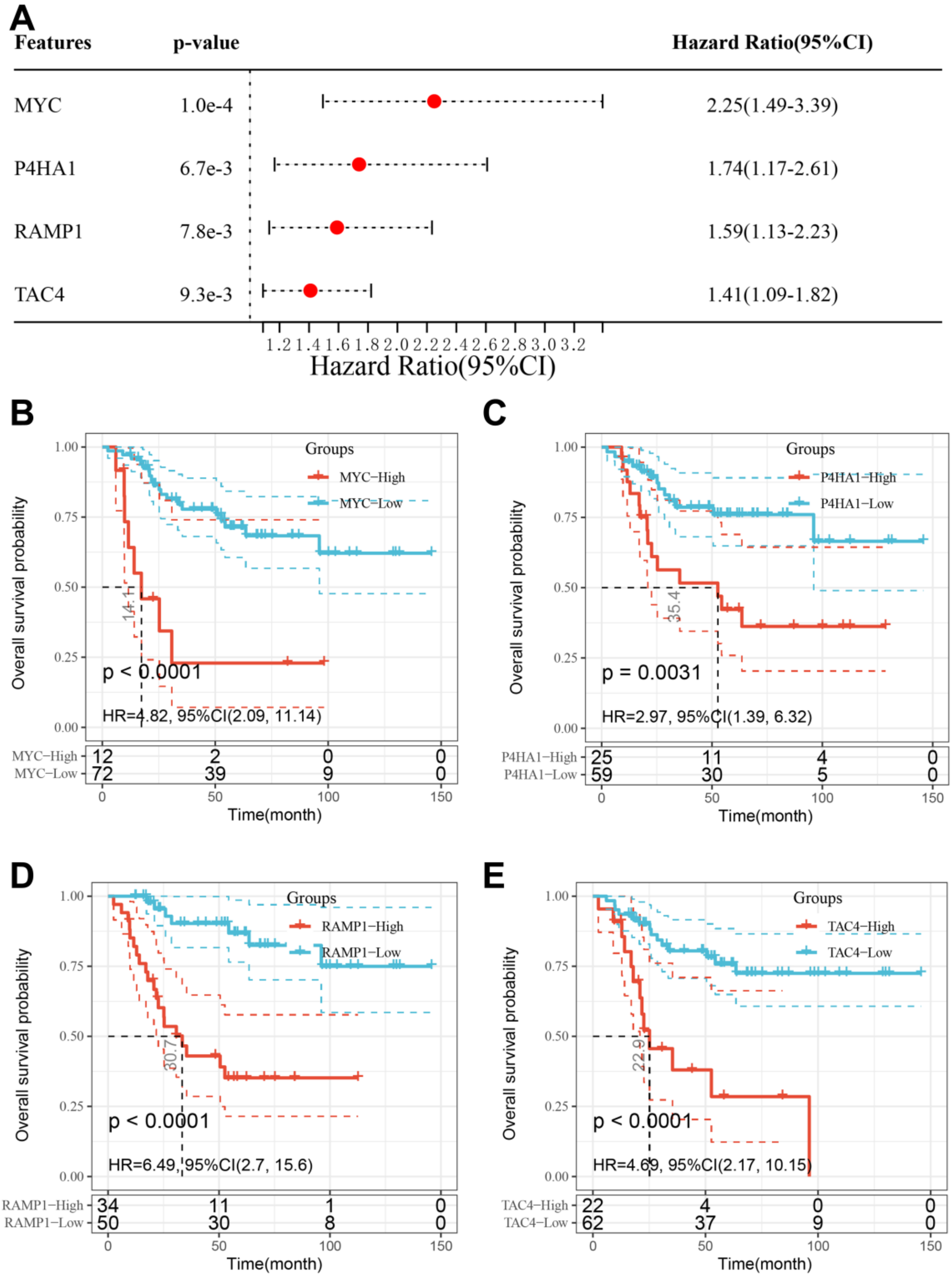

Figure S4. OS prediction value of 4 genes in the TMEindex. A Univariate Cox regression analysis reveals the association of 4 genes in TMEindex with the OS of osteosarcoma. **B-E** Kaplan-Meier curves depict the OS difference between high and low expression of MYC (**B**), P4HA1 (**C**), RAMP1 (**D**) and TAC4 (**E**). Red representing the high expression group and blue representing the low expression group.

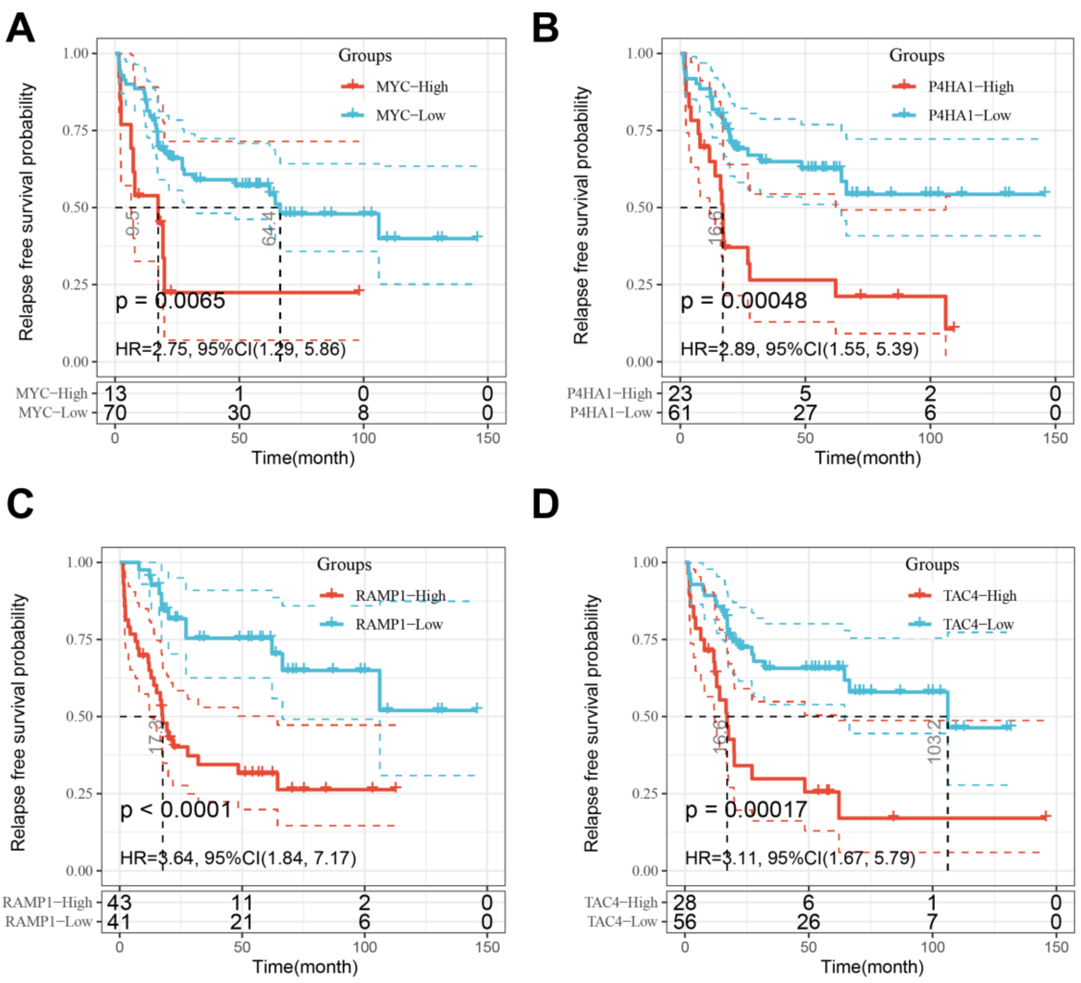

Figure S5. RFS prediction value of 4 genes in the TMEindex. A-D Kaplan-Meier curves depict the RFS difference between high and low expression of MYC (**A**), P4HA1 (**B**), RAMP1 (**C**) and TAC4 (**D**). Red representing the high expression group and blue representing the low expression group.

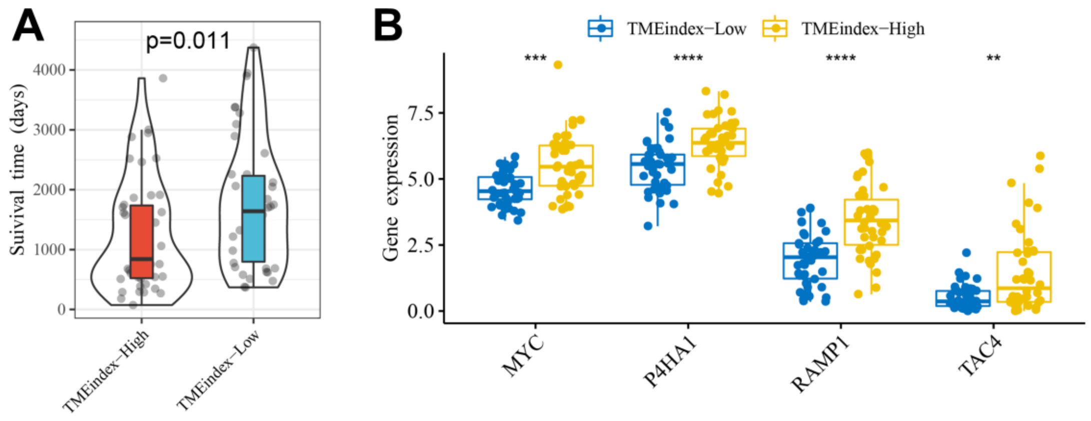

Figure S6. Differences in survival time and TMEindex genes expression between high and low TMEindex groups. A Differences in survival time between high and low TMEindex groups. **B** TMEindex genes expression between high and low TMEindex groups. **** $P < 0.0001$.

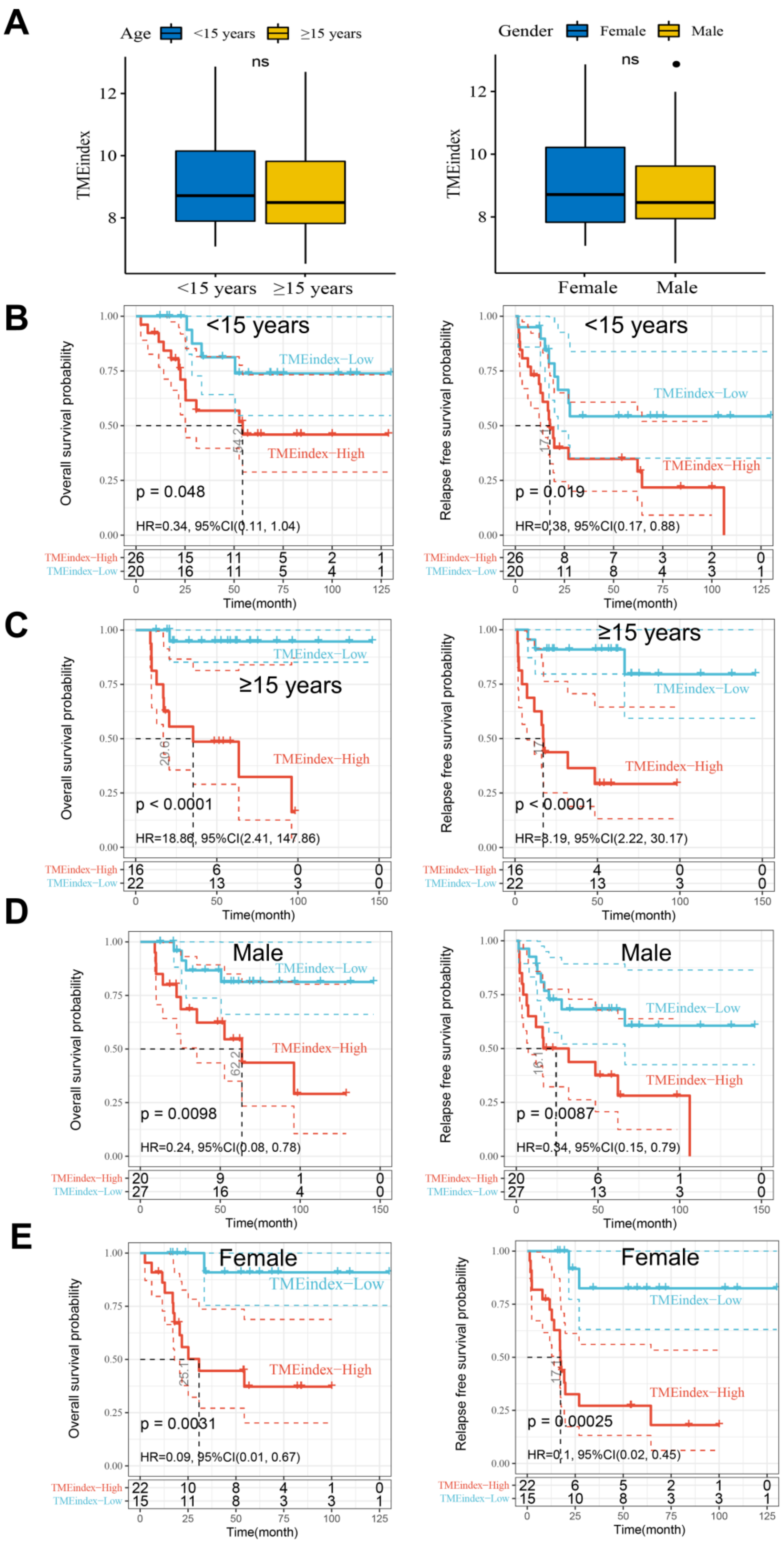
A
Age
<15 years
≥15 years
Gender
Female
Male
ns
TMEindex
B
<15 years
Overall survival probability
Relapse free survival probability
TMEindex−Low
TMEindex−High
p = 0.048
HR=0.34, 95%CI(0.11, 1.04)
p = 0.019
HR=0.38, 95%CI(0.17, 0.88)
Time(month)
C
≥15 years
p < 0.0001
HR=18.86, 95%CI(2.41, 147.86)
HR=8.19, 95%CI(2.22, 30.17)
D
Male
p = 0.0098
HR=0.24, 95%CI(0.08, 0.78)
p = 0.0087
HR=0.34, 95%CI(0.15, 0.79)
E
Female
p = 0.0031
HR=0.09, 95%CI(0.01, 0.67)
p = 0.00025
HR=0.1, 95%CI(0.02, 0.45)

Figure S7. Stratified analysis to further determine the prognostic value of the TMEindex based on the clinical characteristics of patients. A Differences in TMEindex in different age and gender subgroups. **B-E** Stratified OS and RFS analysis performed in patients with different age (**B,C**) and gender (**D,E**). Significance for survival analysis was calculated using a log-rank test, with the red line representing the TMEindex-high group and the blue line representing the TMEindex-low group. The grouping of osteosarcoma samples is shown at the bottom of the charts.

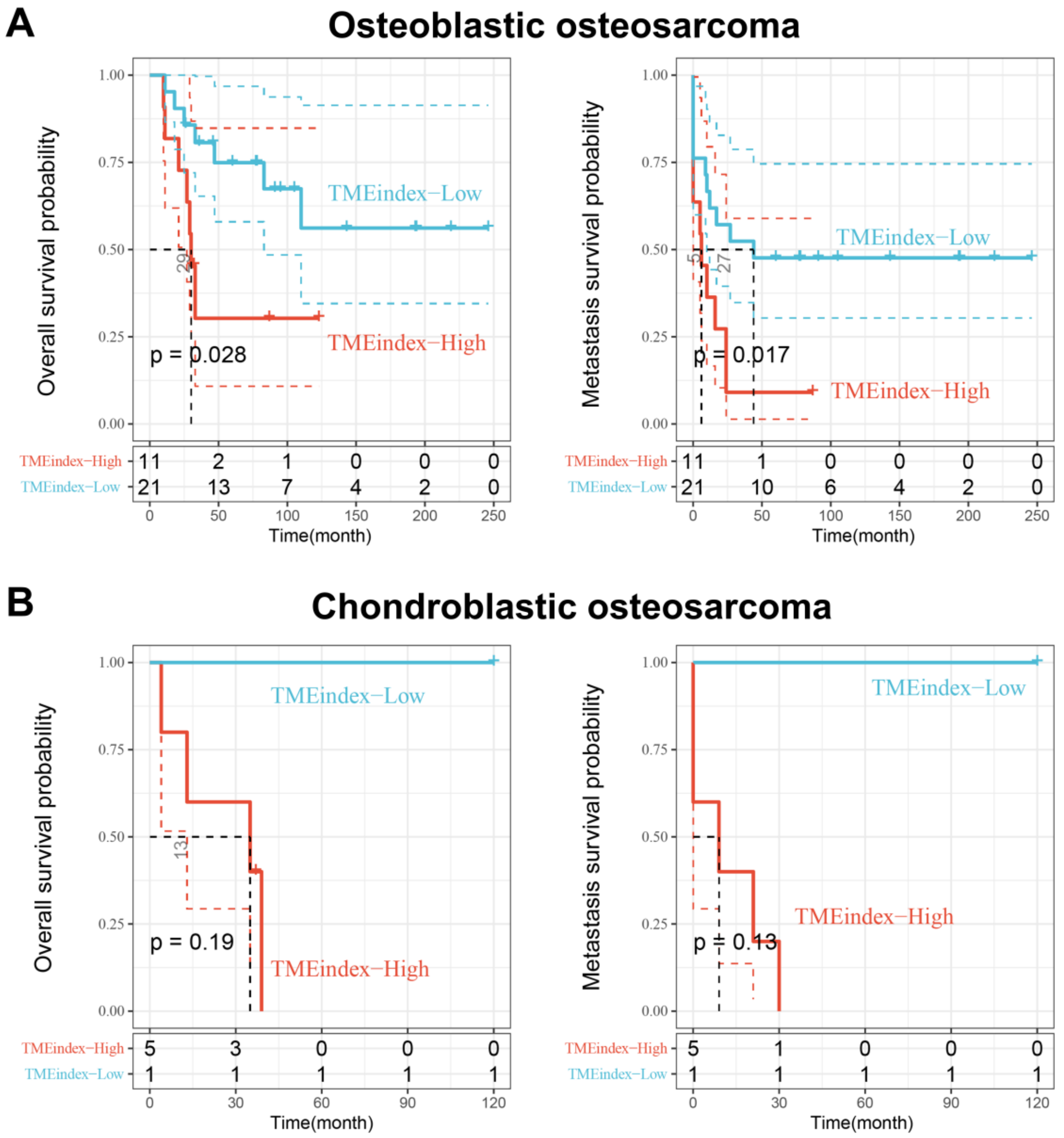

Figure S8. Progonsis prediction value of the TMEindex in osteoblastic and chondroblastic osteosarcoma in the GSE21257 cohort. A Kaplan-Meier curves depict the OS and RFS difference between high and low TMEindex groups in osteoblastic osteosarcoma**. B** Kaplan-Meier curves depict the OS and RFS difference between high and low TMEindex groups in chondroblastic osteosarcoma**.**

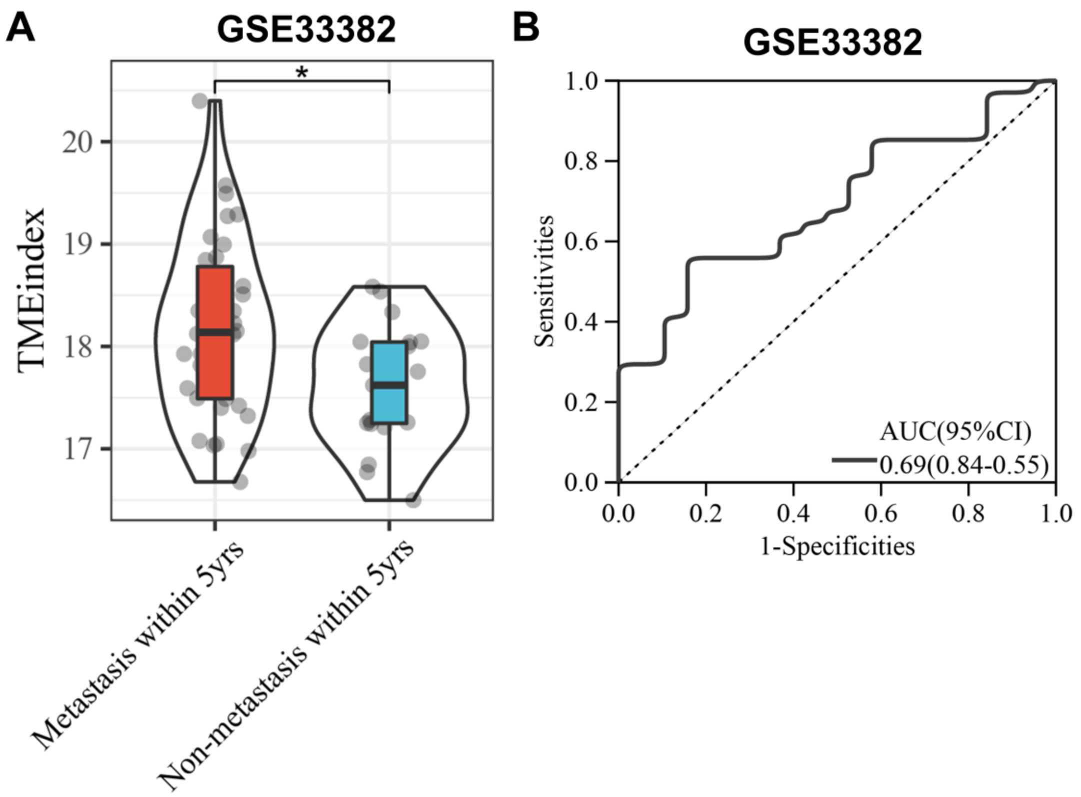

Figure S9. Validation of the prognostic value of the TMEindex in GSE33382. A Difference in TMEindex between patients with metastases within 5 years and those Non-metastases within 5 years. * $P < 0.05$. **B** ROC curve showing the 5 years metastasis status prediction efficiency of the TMEindex in the GSE33382 cohort.

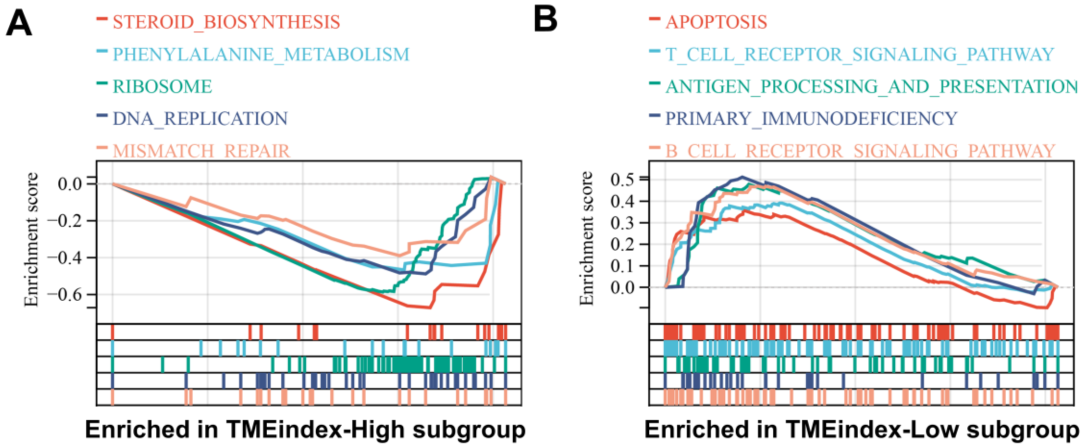

Figure S10. GSEA enrichment plots base on KEGG gene set showing the relatively enriched pathways in TMEindex-high (A) and TMEindex-low (B) groups.

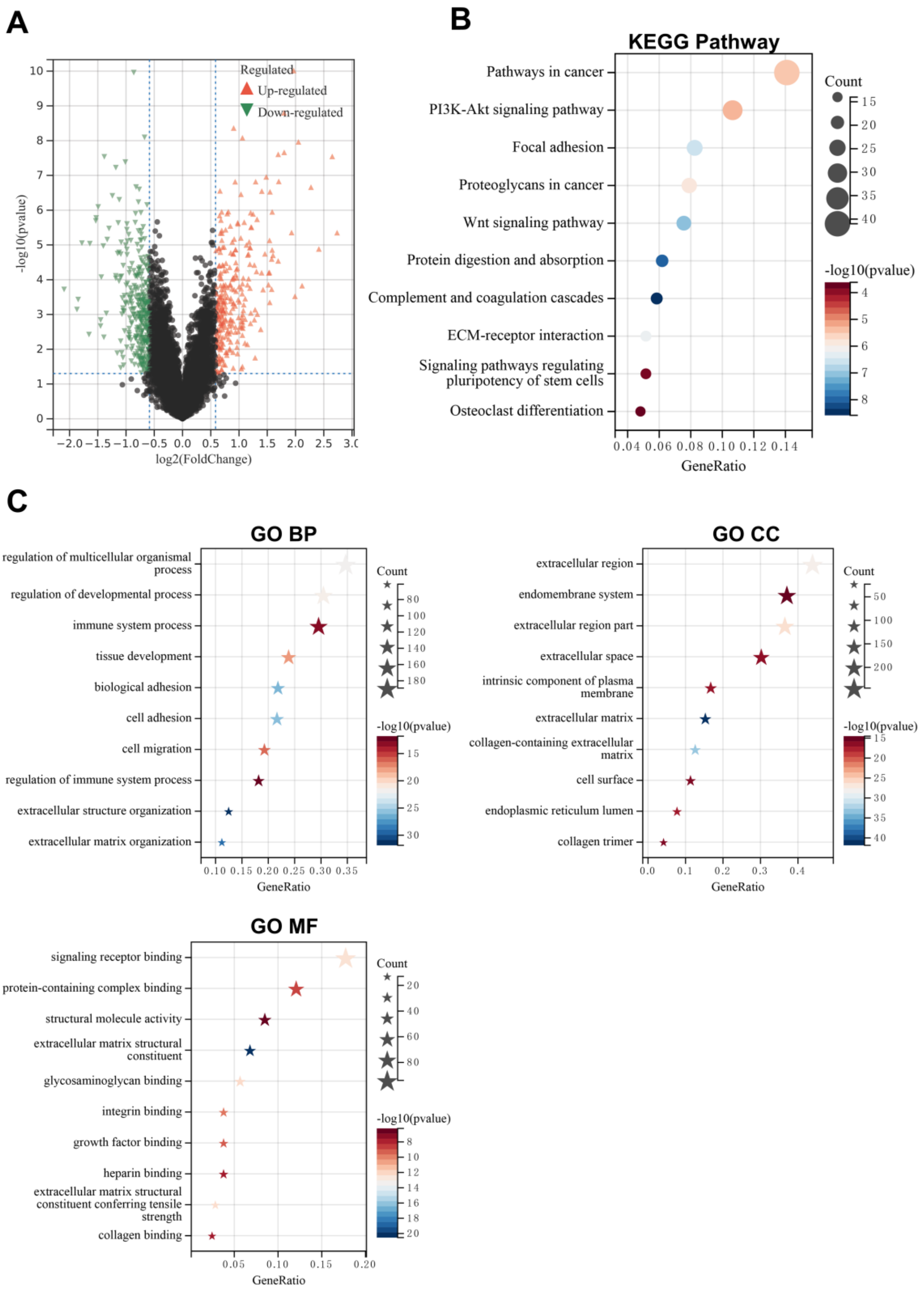

Figure S11. GO enrichment and KEGG pathway analysis of DEGs identified between high and low TMEindex groups. A Volcano plot of DEGs in the TMEindex-high group relative to the TMEindex-low group. Red dots represent up-regulated genes and green represents down-regulated genes. B KEGG pathway terms enriched in DEGs. C GO biological process, cellular component terms and molecular function terms enriched in DEGs.

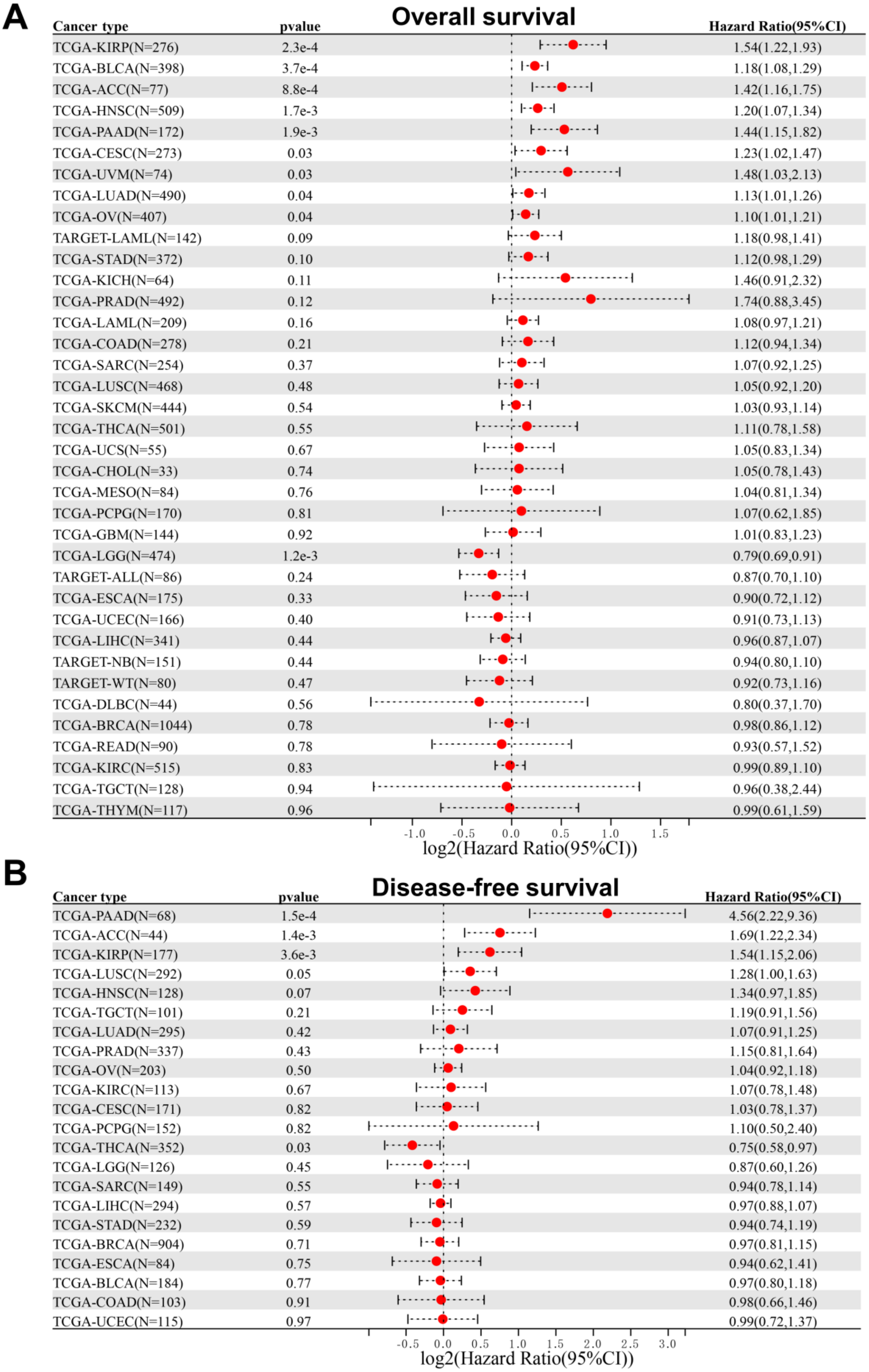
A
Overall survival
Cancer type
pvalue
Hazard Ratio(95%CI)
TCGA-KIRP(N=276) 2.3e-4 1.54(1.22,1.93)
TCGA-BLCA(N=398) 3.7e-4 1.18(1.08,1.29)
TCGA-ACC(N=77) 8.8e-4 1.42(1.16,1.75)
TCGA-HNSC(N=509) 1.7e-3 1.20(1.07,1.34)
TCGA-PAAD(N=172) 1.9e-3 1.44(1.15,1.82)
TCGA-CESC(N=273) 0.03 1.23(1.02,1.47)
TCGA-UVM(N=74) 0.03 1.48(1.03,2.13)
TCGA-LUAD(N=490) 0.04 1.13(1.01,1.26)
TCGA-OV(N=407) 0.04 1.10(1.01,1.21)
TARGET-LAML(N=142) 0.09 1.18(0.98,1.41)
TCGA-STAD(N=372) 0.10 1.12(0.98,1.29)
TCGA-KICH(N=64) 0.11 1.46(0.91,2.32)
TCGA-PRAD(N=492) 0.12 1.74(0.88,3.45)
TCGA-LAML(N=209) 0.16 1.08(0.97,1.21)
TCGA-COAD(N=278) 0.21 1.12(0.94,1.34)
TCGA-SARC(N=254) 0.37 1.07(0.92,1.25)
TCGA-LUSC(N=468) 0.48 1.05(0.92,1.20)
TCGA-SKCM(N=444) 0.54 1.03(0.93,1.14)
TCGA-THCA(N=501) 0.55 1.11(0.78,1.58)
TCGA-UCS(N=55) 0.67 1.05(0.83,1.34)
TCGA-CHOL(N=33) 0.74 1.05(0.78,1.43)
TCGA-MESO(N=84) 0.76 1.04(0.81,1.34)
TCGA-PCPG(N=170) 0.81 1.07(0.62,1.85)
TCGA-GBM(N=144) 0.92 1.01(0.83,1.23)
TCGA-LGG(N=474) 1.2e-3 0.79(0.69,0.91)
TARGET-ALL(N=86) 0.24 0.87(0.70,1.10)
TCGA-ESCA(N=175) 0.33 0.90(0.72,1.12)
TCGA-UCEC(N=166) 0.40 0.91(0.73,1.13)
TCGA-LIHC(N=341) 0.44 0.96(0.87,1.07)
TARGET-NB(N=151) 0.44 0.94(0.80,1.10)
TARGET-WT(N=80) 0.47 0.92(0.73,1.16)
TCGA-DLBC(N=44) 0.56 0.80(0.37,1.70)
TCGA-BRCA(N=1044) 0.78 0.98(0.86,1.12)
TCGA-READ(N=90) 0.78 0.93(0.57,1.52)
TCGA-KIRC(N=515) 0.83 0.99(0.89,1.10)
TCGA-TGCT(N=128) 0.94 0.96(0.38,2.44)
TCGA-THYM(N=117) 0.96 0.99(0.61,1.59)
-1.0 -0.5 0.0 0.5 1.0 1.5
log2(Hazard Ratio(95%CI))
B
Disease-free survival
Cancer type
pvalue
Hazard Ratio(95%CI)
TCGA-PAAD(N=68) 1.5e-4 4.56(2.22,9.36)
TCGA-ACC(N=44) 1.4e-3 1.69(1.22,2.34)
TCGA-KIRP(N=177) 3.6e-3 1.54(1.15,2.06)
TCGA-LUSC(N=292) 0.05 1.28(1.00,1.63)
TCGA-HNSC(N=128) 0.07 1.34(0.97,1.85)
TCGA-TGCT(N=101) 0.21 1.19(0.91,1.56)
TCGA-LUAD(N=295) 0.42 1.07(0.91,1.25)
TCGA-PRAD(N=337) 0.43 1.15(0.81,1.64)
TCGA-OV(N=203) 0.50 1.04(0.92,1.18)
TCGA-KIRC(N=113) 0.67 1.07(0.78,1.48)
TCGA-CESC(N=171) 0.82 1.03(0.78,1.37)
TCGA-PCPG(N=152) 0.82 1.10(0.50,2.40)
TCGA-THCA(N=352) 0.03 0.75(0.58,0.97)
TCGA-LGG(N=126) 0.45 0.87(0.60,1.26)
TCGA-SARC(N=149) 0.55 0.94(0.78,1.14)
TCGA-LIHC(N=294) 0.57 0.97(0.88,1.07)
TCGA-STAD(N=232) 0.59 0.94(0.74,1.19)
TCGA-BRCA(N=904) 0.71 0.97(0.81,1.15)
TCGA-ESCA(N=84) 0.75 0.94(0.62,1.41)
TCGA-BLCA(N=184) 0.77 0.97(0.80,1.18)
TCGA-COAD(N=103) 0.91 0.98(0.66,1.46)
TCGA-UCEC(N=115) 0.97 0.99(0.72,1.37)
-0.5 0.0 0.5 1.0 1.5 2.0 2.5 3.0
log2(Hazard Ratio(95%CI))

Figure S12. Pan-cancer analysis of the prognostic predictive value of TMEindex. A Univariate Cox regression analysis reveals the association of TMEindex with the OS of 37 tumor types. **B** Univariate Cox regression analysis reveals the association of TMEindex with the DFS of 22 tumor types.

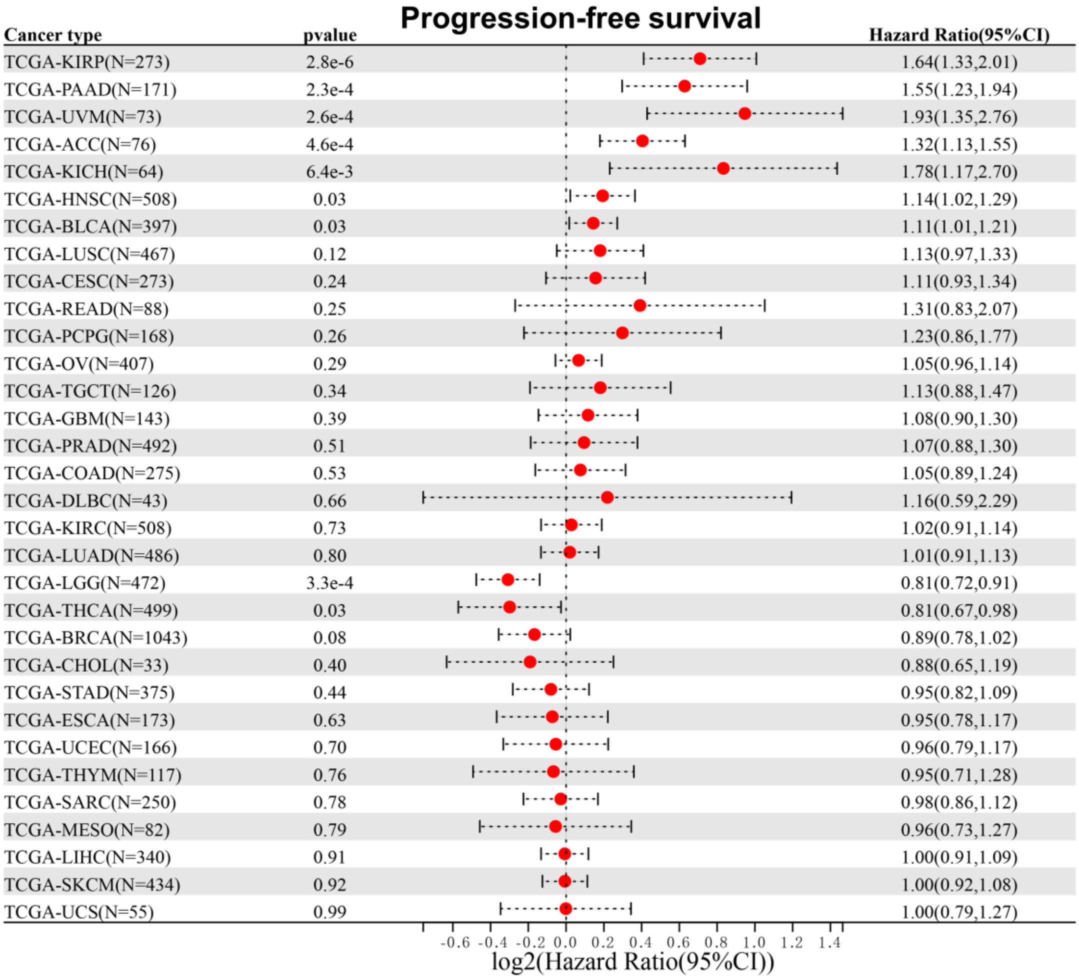

Figure S13. Univariate Cox regression analysis reveals the association of TMEindex with the PFS of 32 tumor types.
